# Greenhouse gas removal in agricultural peatland via raised water levels and soil amendment

**DOI:** 10.1007/s42773-024-00422-2

**Published:** 2025-02-21

**Authors:** Peduruhewa H. Jeewani, Robert W. Brown, Jennifer M. Rhymes, Niall P. McNamara, David R. Chadwick, Davey L. Jones, Chris D. Evans

**Affiliations:** 1 School of Environmental and Natural Sciences, Bangor University, Bangor, Gwynedd LL57 2UW UK; 2 UK Centre for Ecology & Hydrology, Bangor, Gwynedd LL57 2UW UK; 3 UK Centre for Ecology & Hydrology, Lancaster, LA1 4AP UK

**Keywords:** Carbon sequestration, Biochar, Sustainable agriculture, Methane, Carbon dioxide, Peat

## Abstract

Peatlands are an important natural store of carbon (C). Drainage of lowland peatlands for agriculture and the subsequent loss of anaerobic conditions had turned these C stores into major emitters of greenhouse gases (GHGs). Practical management strategies are needed to reduce these emissions, and ideally to reverse them to achieve net GHG removal (GGR). Here we show that a combination of enhanced C input as recalcitrant organic matter, CH_4_ suppression by addition of terminal electron acceptors, and suppression of decomposition by raising water levels has the potential to achieve GGR in agricultural peat. We measured GHG (CO_2_, N_2_O, and CH_4_) fluxes for 1 year with intensive sampling (6 times within the first 56 days) followed by monthly sampling in outdoor mesocosms with high (0 cm) and low (− 40 cm) water table treatments and five contrasting organic amendments (*Miscanthus*-derived biochar, *Miscanthus* chip, paper waste, biosolids, and barley straw) were applied to high water table cores, with and without iron sulphate (FeSO_4_). Biochar produced the strongest net soil C gain, suppressing both peat decomposition and CH_4_ emissions. No other organic amendment generated similar GGR, due to higher decomposition and CH_4_ production. FeSO_4_ application further suppressed CO_2_ and CH_4_ release following biochar addition. While we did not account for life-cycle emissions of biochar production, or its longer-term stability, our results suggest that biochar addition to re-wetted peatlands could be an effective climate mitigation strategy.

**Supplementary Information:**

The online version contains supplementary material available at 10.1007/s42773-024-00422-2.

## Highlights

- Biochar amendment to rewetted agricultural peat soil is an effective greenhouse gas removal strategy.
- Addition of organic amendments with low C:N ratio (e.g. straw) increased CO_2_ emissions due to input of labile C.
- A high-water table increased CH_4_ emissions, but suppressed N_2_O emissions and CO_2_ emissions, cumulatively.
- FeSO_4_ addition reduced CH_4_ emissions by the provision of alternate electron acceptors.

**Supplementary Information:**

The online version contains supplementary material available at 10.1007/s42773-024-00422-2.

## Introduction

Peatlands cover ca. 4 million km^2^ (3%) of the earth land surface and store up to 550 Gt of carbon (C) globally, representing the largest terrestrial C pool (Unep 2022; Minasny et al. 2023). This large C stock in peatlands is caused by slow rates of organic matter decomposition arising from the anaerobic conditions and C-rich plant inputs (Fenner and Freeman 2011; Mitsch et al. 2013; Kirk et al. 2015). However, ca. 20% of the world peatlands are now exploited for agricultural use (Dise 2009; Bonn et al. 2016; Kwon et al. 2022). Drainage and subsequent aeration of these peats to improve agricultural productivity promotes microbial activity and the loss of soil organic C (SOC), turning peatlands from net C sinks to major sources of greenhouse gas (GHG) emissions (Bonn et al. 2016; Leifeld and Menichetti 2018; Freeman et al. 2022). Agricultural peat drainage is estimated to release 645 t C yr⁻^1^ (ranging from 401 to 1025 t C yr⁻^1^) through soil respiration, accounting for approximately 5% of global annual anthropogenic carbon dioxide (CO_2_) emissions (Evans et al. 2021; Ma et al. 2022). Practical and cost-effective strategies are therefore urgently needed to mitigate C loss from agricultural peatlands (Freeman et al. 2022).

According to the Paris Agreement, global leaders committed to progressively more ambitious actions to reduce GHG emissions and limit global warming (Fuss et al. 2020). To achieve current targets, however, new approaches will be required to capture and store CO_2_ (Fuss et al. 2020). Nature-based solutions have significant potential to contribute to CO_2_ removal, via enhanced C sequestration into biomass or soil, with an estimated maximum mitigation potential of up to 23.8 Pg CO_2_eq yr^−1^ in the short term (to 2030) (Griscom et al. 2017). Peatlands appear obvious candidates for nature-based CO_2_ capture and storage, given their intrinsic capacity to continuously accumulate and retain C via peat formation over millennia. However, the combination of relatively slow C sequestration rates in natural peatlands, and the risk of high methane (CH_4_) emissions from re-wetted peatlands (effectively cancelling out the benefits of CO_2_ sequestration, at least in the short term), has led to their potential for net greenhouse gas removal (GGR) being largely discounted (Knox et al. 2015; Bui et al. 2018; Gao et al. 2018). Identifying practical, environmentally sound, cost-effective, and socially acceptable GGR methods for restoring agricultural peatlands is an urgent need.

Many studies have shown that in order to mitigate CO_2_ emissions from peat soils, water tables need to be raised, resulting in slower SOC decomposition and CO_2_ release by restoring anaerobic conditions (Bonn et al. 2016; Evans et al. 2021; Ma et al. 2022). However, the proximity of the water level to the surface of soil have a major effect on the balance of CO_2_, CH_4_, and nitrous oxide (N_2_O) release, potentially leading to negative consequences such as increased CH_4_ or N_2_O emissions, which have higher radiative forcing impact than CO_2_ over shorter timescales (Waddington and Day 2007; Kandel et al. 2019, 2020). To date, the re-wetting and restoration of peatlands have largely focused on reducing or halting emissions, rather than on capturing new C or turning peatlands into net GHG sinks.

To overcome these issues and deliver significant GGR through peatland re-wetting, additional interventions may be needed, both to enhance the rate of C input and to avoid increased emissions of CH_4_ and/or N_2_O. The addition of organic amendments in combination with raised water tables has the potential to deliver these objectives, but remains poorly understood (Evans et al. 2021). The GHG balance is likely to be dependent on moisture status of soil, the quality and quantity of C and N added, and its subsequent microbial availability (Butterbach-Bahl et al. 2013; Kandel et al. 2019). For example the addition of N-rich substrates may promote N_2_O emissions, through either nitrification or denitrification depending on the soil oxygen status (Butterbach-Bahl et al. 2013).

The capacity of biochar to contribute to climate change mitigation and enhance soil health has garnered significant global attention. The average estimated negative emission potential of biochar is 1.07 GtCO_2_ per year under sustainable scenarios, with a range of 0.68–1.46 GtCO_2_ per year (Deng et al. 2024). Biochar is pyrolyzed product of organic matter, and is generally considered a durable store of C. During pyrolysis, aromatic structures are formed, rendering a large proportion of the C in the material biologically unavailable when applied to land (Liu et al. 2011; He et al. 2017). Beyond C storage of the material itself, a number of GHG-related co-benefits to applying biochar have been suggested. These include elevated soil pH on application, stimulating the N_2_O reduction of denitrifying bacterial communities and thereby reducing soil N_2_O emissions, and enhanced soil aeration leading to increased CH_4_ oxidation (Liu et al. 2011; He et al. 2017; Abagandura et al. 2019). Furthermore, biochar created from woody biomass feedstock has a high porosity, facilitating the absorption of soil C onto its surface or within its pores, thereby restricting C mineralization (Liu et al. 2011).

The tendency of wet peatlands to generate high CH_4_ emissions is related to the lack of terminal electron acceptors (notably oxygen) in waterlogged soils, which permits the less energetically favorable process of methanogenesis to occur where organic substrate is present.

Small-scale experiments have suggested that the presence of alternative terminal electron acceptors, such as sulphate (SO_4_^2−^) and iron (III) (Fe^3+^), may therefore limit methanogenesis, and also denitrification (Wen et al. 2019). Greater SO_4_^2−^ abundance will benefit SO_4_^2−^-reducing microorganisms, which are known to suppress the activity of methanogenic microorganisms in wetlands by outcompeting them for labile substrate (Pester et al. 2012). Iron is also known to play a role in SOC stability and C storage (Lalonde et al. 2012; Li et al. 2012; Kramer and Chadwick 2018), particularly in fluctuating redox environments that involve the mobilization and stabilization of C.

To our knowledge, no previous studies have explored the interactive effects of organic and inorganic amendments and water table manipulation on GHG emissions from lowland agricultural peatland soils. We hypothesized that these management interventions may provide new tools for promoting effective C storage and GHG removal in peat soils. The aims of this study were to quantify how contrasting organic and inorganic amendments and water table depth (high vs low) affect GHG emissions from cultivated lowland peat soils. We aimed to identify amendments that could increase soil C (i.e. sequester CO_2_) without offsetting GHG emissions by accelerating decomposition of existing SOC or enhancing CH_4_ or N_2_O emissions. We hypothesized that: (1) adding high C:N ratio amendments under high water table conditions would promote C storage and immobilize inorganic N; (2) adding low C:N ratio organic amendments would increase CO_2_ emissions due to input of labile C and increase N_2_O emissions due to increased mineralization; (3) high-water table management with recalcitrant high C substrate addition (biochar) would decrease CH_4_ and suppress N_2_O and CO_2_ relative to the control; (4) adding FeSO_4_ to high water table to agricultural peat soil would reduce CH_4_ emissions by the provision of alternative electron acceptors.

## Materials and methods

### Study site and experimental design

Intact soil mesocosms were collected from a commercial agricultural lowland peat field at Lapwing estate, Doncaster, DN10 4SN, UK (53°27’N 00′54'W) in May 2022. The site consists of a flat and drained lowland fen (ca. 40–80 cm organic layer overlying a mineral soil). The area was first drained in the 1600s and has been subject to severe and ongoing peat oxidation and wastage since this time. During the last 20 years, the study field has been under intensive agricultural crop (e.g. *Brassica*) and grass rotation. The area has a mean annual temperature of 10.3 °C and annual mean rainfall of 1162 mm, and the soil type is classified as an Ombric Sapric Histosol (Schad, 2016). To maintain soil structure and prevent compaction, PVC pipes (⌀ = 20 cm, height = 60 cm) with a sharpened basal edge were inserted into the unvegetated soil to 50 cm depth. The intact peat cores were mechanically excavated and transported to Bangor University, where they were kept outdoors throughout the entire 365-day experimental period.

### Experimental design

The mesocosm experiment comprised of 14 treatments (*n* = 4 replicates per treatment), including five organic amendments with a gradient of C: N ratios. The treatments included: (i) peat cores with a low water table; −40 cm below the soil surface (LWT), (ii) peat cores with a high-water table; at the soil surface (0 cm; HWT). All subsequent organic amendments were applied to the HWT treatments only; (a) *Miscanthus giganteus* derived chip (size ranges from 1 to 2 cm; C:N ratio = 96), (b) pyrolyzed *M. giganteus* chip (size ranges from 1 to 2 cm) (biochar; pyrolysed at 450 °C, 30 min using the muffle furnace; C: N ratio = 258), (c) paper waste of commercial paper production from Ahlstrom Chirnside Ltd, Manchester, UK (C:N ratio = 155), (d) barley straw (*Hordeum vulgare* L.) (C: N ratio = 63), and (e) anaerobically digested biosolids from a large urban wastewater treatment plant (C: N ratio = 10). These treatments represented a range of C:N ratios from labile (low C:N, biosolids) to recalcitrant (high C:N, biochar), indicative of different qualities of organic matter input (Ghosh and Leff 2013). Each treatment was evaluated either in the presence or absence of added FeSO_4_. All organic substrates were applied at a loading rate of 20 t C ha^−1^ (Jones et al. 2012; Pandit et al. 2018), and the FeSO_4_ at a rate of 0.5 t ha^−1^ (Wen et al. 2019). Both the organic amendments and the FeSO_4_ were carefully mixed, by hand, into the top 10 cm of soil to simulate field-based mechanical harrowing. The experiment was started in late May 2022 and continued for a year. Initially, we conducted intensive sampling on days 3, 5, 9, and 14, then biweekly until day 56 at which point sampling continued monthly up to 12 months. The intensity of the sampling regime was chosen in order to ensure that the GHG fluxes from amendments were adequately reflected.

Each mesocosm was then placed into an outer container with drainage holes drilled to maintain the high or low water table level (Supplementary Fig. 1). The mesocosms were left for 3 days to equilibrate at their new respective water tables before the experiment started. Throughout the experiment the water table was maintained either through natural rainfall, or during dry periods via the addition of tap water. Water table depths were selected to achieve predominantly anaerobic conditions in the "re-wetted" HWT treatments, and aerobic conditions in the LWT control treatment, representing "business as usual" drainage-based agricultural management.

### Basic characteristics of soil and organic amendments

Initial soil characteristics were quantified in three section (0–10, 10–30, and 30–50 cm depth) at the start of the experiment. The pH and electrical conductivity (EC) of soil and organic amendments (sieved to < 2 cm) were analyzed on 1:2.5 (w/v) soil-to-water solutions using Sension + MM150 Portable Multi-Parameter Meter (Hach UK, Manchester, UK). The bulk density was determined using the fixed volume ring method (Blake and Hartge 1986). Total C (TC) and N (TN) of soil and organic amendments were measured from oven-dried (80 °C, 16 h) and ground samples using a TruSpec^®^ CN Analyzer (Leco Corp., St. Joseph, MI). Soil organic matter (SOM) was determined by calculating the loss‐on‐ignition in a muffle furnace (450 °C, 16 h). Dissolved organic carbon (DOC) and total dissolved N (TDN) were measured using 1:5 (*w*/*v*) soil-to-0.5 M K_2_SO_4_ extracts on a Multi N/C 2100/2100 analyzer (Analytik Jena AG, Jena, Germany). Available ammonium (NH_4_^+^) (Mulvaney 1996), nitrate (NO_3_^–^) (Miranda et al. 2001), phosphate (PO_4_^3−^) (Murphy and Riley 1962) and SO_4_^2−^ (Rowell 2014) concentration of soil solutions and amendments were extracted in 1:5 (*w*/*v*) soil-to-deionized H_2_O suspensions measured by spectrophotometry on a Power Wave‐XS microplate reader (BioTek Instruments Inc., USA) using the colorimetric methods (Bradfield and Cooke 1985). Soil and organic amendment characteristics were shown in Supplementary Table 1. The biochar was assessed for atomic H/C ratio and the stable polyaromatic carbon fraction determined by Hypy test (Ascough et al. 2009) (Supplementary Table 2).

### Soil GHG flux measurements and calculations

During each sampling event, gas-tight PVC lids (20 cm inner diameter, 4 cm height) sealed with a Suba-Seal^®^ gas sampling lids (Sigma-Aldrich Ltd., Poole, UK) were placed onto each core, to create a sealed headspace (3145 cm^3^). After lid closure, 20 ml headspace gas samples were taken using an air‐tight 20 ml plastic syringe after 0-, 20-, and 40-min. Gas samples were introduced into pre-evacuated 20 ml vials sealed with rubber septa (QUMA Electronik & Analytik GmbH, Wuppertal, Germany). The concentrations of CO_2_, CH_4_, and N_2_O were subsequently measured using a Gas Chromatograph with a TurboMatrix 110 autosampler (PerkinElmer Corp, Waltham, CT, USA). A split injector allowed the sample to be passed through two Elite-Q mega bore columns, with one connected to an ECD for N_2_O determination, and the other to an FID for CH_4_ and CO_2_ (via a methaniser) determination.

Greenhouse gas emissions were calculated by subtracting the gas concentrations at time 0 from those measured 40 min later, with adjustments made for temperature and the ratio of chamber volume to soil surface area (Sánchez-Rodríguez et al. 2019).


1


where *R*_*i*−1_ and *R*_*i*_ are the rate of GHG flux in the *i*-1 and *i*th sampling, *D*_i_ is the number of days between *i*-1 and *i*th sampling and *n* is the number of sampling times. To allow comparison among treatments, GHG emissions were converted to CO_2_ equivalents (CO_2_eq) based on 100‐yr global warming potential conversion factors of 265 for N_2_O and 28 for CH_4_ (IPCC 2023).

2 $${\text{Total GHG emissions (CO}}_{{2 }} {\text{equivalent) = CO}}_{{2}} { + (265 } \times {\text{ N}}_{{2}} {\text{O) + (28 }} \times {\text{ CH}}_{{4}} )$$

C balance of the mesocosm was calculated as follows:

3 $$\text{Original C content}={\sum }_{\text{i}=1}^{4}{C}_{\text{i}}\times {P}_{\text{i}}$$

where, *C*_i_ is the C content of each treatment (t C ha^−1^) and *P*_i_ is proportion of the whole soil mass represented by each treatment.

4 $$\text{Total C content}=\text{Original C content}+\text{C addition}$$

5 $$\text{C loss}=\text{Cumulative emissions of }{\text{CO}}_{2}+\text{Cumulative emissions of }{\text{CH}}_{4}$$

Assuming no losses of DOC, C losses from the mesocosms were calculated by summing the total CO_2_ and CH_4_ fluxes. The difference between original C content and C loss was defined as C storage, calculated according to the following equation:

6 $$\text{C storage}=\text{Total C content}-\text{C loss}$$

### Statistical analysis

Differences in soil variables between the treatments were analysed with one-way and two-way ANOVA using SPSS 24 package (SPSS, Chicago, IL, USA). Data were checked for normality and homogeneity by the tests of Shapiro–Wilk and Levene, respectively. If conditions were not met, the data were either log_10_ or square root transformed prior to analysis. A Tukey Post-hoc test was performed to determine differences between treatments on each sampling date. The t tests of students were conducted to analyze the differences between treatments using the R software. Values of *P* < 0.05 were considered statistically significant. Only statistically significant results were discussed. Visualization of gas fluxes were performed in Origin 2022 (Origin Lab Corp, USA).

## Results

### Effect of water table depth, C amendment and FeSO_4_ on greenhouse gas emissions

**Fig. 1:**
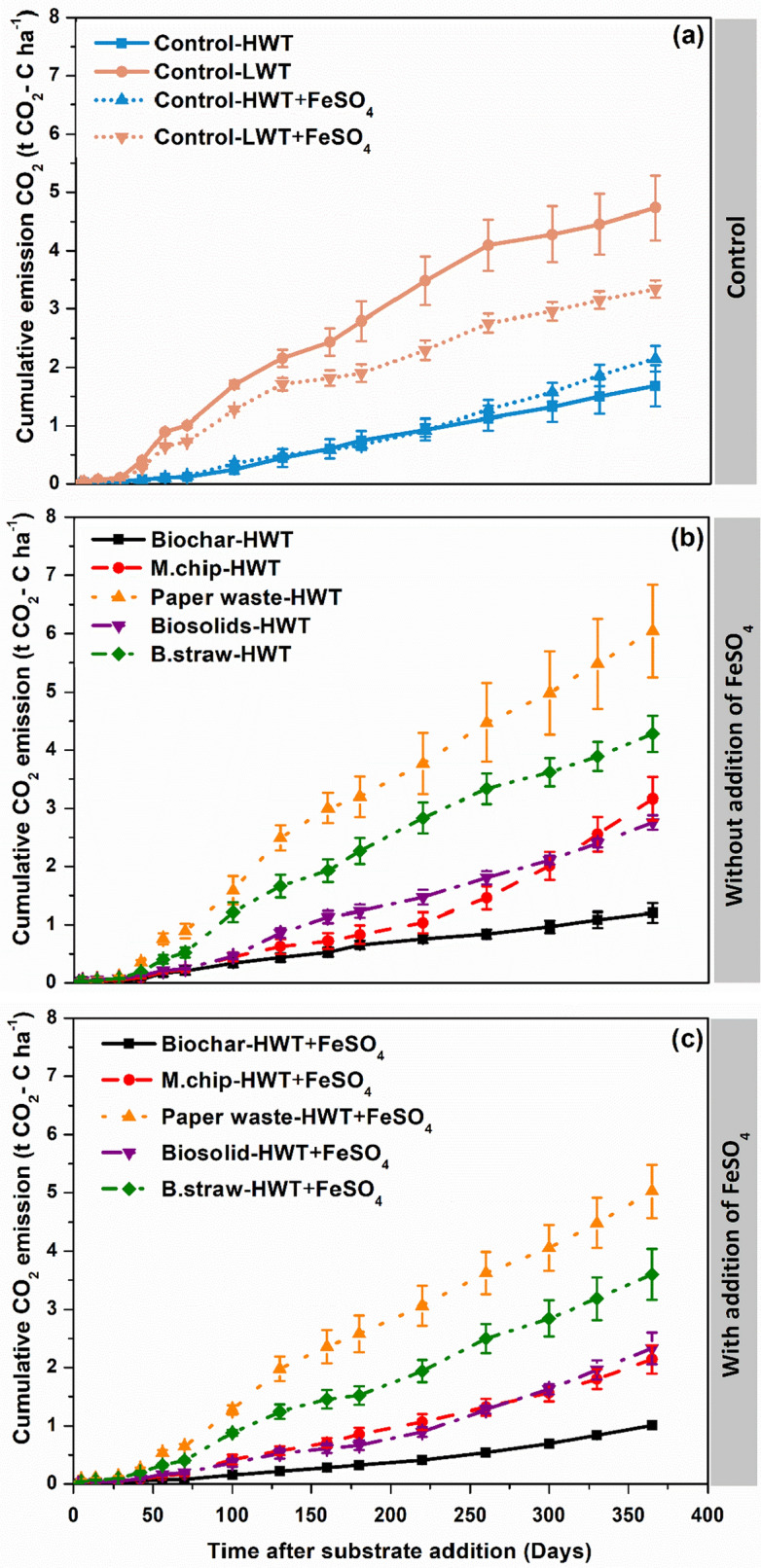
Effect of organic carbon amendments and FeSO_4_ addition on cumulative soil CO_2_ fluxes from an agricultural peat soil. The experiment had two control consisting of a low water table (LWT) treatment and high water table (HWT) without C amendement with and without FeSO_4_ addition. The carbon amendments included *Miscanthus* biochar (Biochar), *Miscanthus* chips (M.chip), paper waste, biosolids and barley straw (B.straw). Values represent means ± standard errors (*n* = 4)

During the 365-day experimental period, cumulative CO_2_ emissions in the high water table control (Control-HWT) were 2.9-fold lower than in the Control-LWT ‘business as usual’ treatment (1.6 t versus 4.7 t CO_2_ ha^−1^ yr^−1^) (*P* = 0.0053) (Fig. 1a, b, Table 1). Cumulative CO_2_ emissions in the organic amendments ranged from 1.2 to 6.04 t CO_2_ ha^−1^ yr^−1^ with the lowest emission observed in the Biochar-HWT treatment and highest in the Paperwaste-HWT treatment (Fig. 1a) (*P* = 0.008). The addition of FeSO_4_ reduced cumulative CO_2_ emissions in all treatments compared to those without FeSO_4_ (*P* = 0.08). In the Fe-amended treatments, cumulative CO_2_ emissions ranged from 1.01 to 5.02 t CO_2_ ha^−1^ yr^−1^, with the lowest emissions observed in the Biochar-HWT + FeSO_4_ treatment and the highest in the Paperwaste-HWT + FeSO_4_ treatment (*P* = 0.003) (Fig. 1a).

**Table 1:** Carbon and greenhouse gas balance with respect to organic C amendment and FeSO_4_ addition in an agricultural peat soil

Treatments	Biomass C added (t C ha^−1^)	Biomass C added (t CO_2_ ha^−1^)	Cumulative CO_2_ flux (t CO_2_e ha ^−1^ yr^−1^)	Cumulative CH_4_ flux (t CO_2_e ha ^−1^ yr^−1^)	Cumulative N_2_O flux (t CO_2_e ha ^−1^ yr^−1^)	C balance (t C ha^−1^)	GHG balance (t CO_2_e ha ^−1^ yr^−1^)	Net CO_2_ difference vs Control-LWT (t CO_2_e ha ^−1^ yr^−1^)	Net GHG difference vs Control-LWT (t CO_2_e ha ^−1^ yr^−1^)
Biochar-HWT + FeSO_4_	20	73.3	3.7	1.9	2.6	− 18.9	− 65.1	− 24.1	− 99.8
Biochar-HWT	20	73.3	4.4	6.9	2.6	− 18.6	− 59.5	− 23.7	− 94.2
Biosolids-HWT + FeSO_4_	20	73.3	8.6	15.8	3.2	− 17.2	− 45.8	− 22.4	− 80.5
M. chip-HWT + FeSO_4_	20	73.3	7.8	21.6	1.1	− 17.3	− 42.8	− 22.4	− 77.5
M. chip-HWT	20	73.3	11.6	28.4	2.5	− 16.1	− 30.8	− 21.2	− 65.5
B. Straw-HWT	20	73.3	15.7	46.7	1.9	− 14.5	− 9.0	− 19.6	− 43.7
B. Straw-HWT + FeSO_4_	20	73.3	13.2	63.1	1.7	− 14.7	4.7	− 19.8	− 30.1
Biosolids-HWT	20	73.3	10.1	66.5	1.9	− 15.5	5.2	− 20.6	− 29.6
Paper waste-HWT + FeSO_4_	20	73.3	18.6	60.5	2.4	− 13.3	8.1	− 18.5	− 26.7
Control-HWT + FeSO_4_	0	0.0	7.9	15.2	3.5	2.6	26.6	− 2.6	− 8.2
Control-LWT + FeSO_4_	0	0.0	12.3	8.6	5.9	3.6	26.7	− 1.5	− 8.0
Control-LWT	0	0.0	17.4	14.1	3.3	5.1	34.7	0.0	0.0
Control-HWT	0	0.0	6.2	27.6	3.9	2.4	37.6	− 2.7	2.9
Paper waste-HWT	20	73.3	22.2	136.8	1.4	− 10.3	87.1	− 15.4	52.4

The C amendments included *Miscanthus* biochar, *Miscanthus* chips (M.chip), paper waste, biosolids barley straw (B.straw). The experiment had two controls consisting of a low water table (LWT) treatment and high water table (HWT) without C amendment, each with and without FeSO_4_ addition. Values represent mean ± standard errors (*n* = 4). Emissions of CH_4_ and N_2_O were converted to CO_2_ equivalents based on their respective 100-year global warming potentials (IPCC Assessment Report: Climate Change, 2023)

**Fig. 2:**
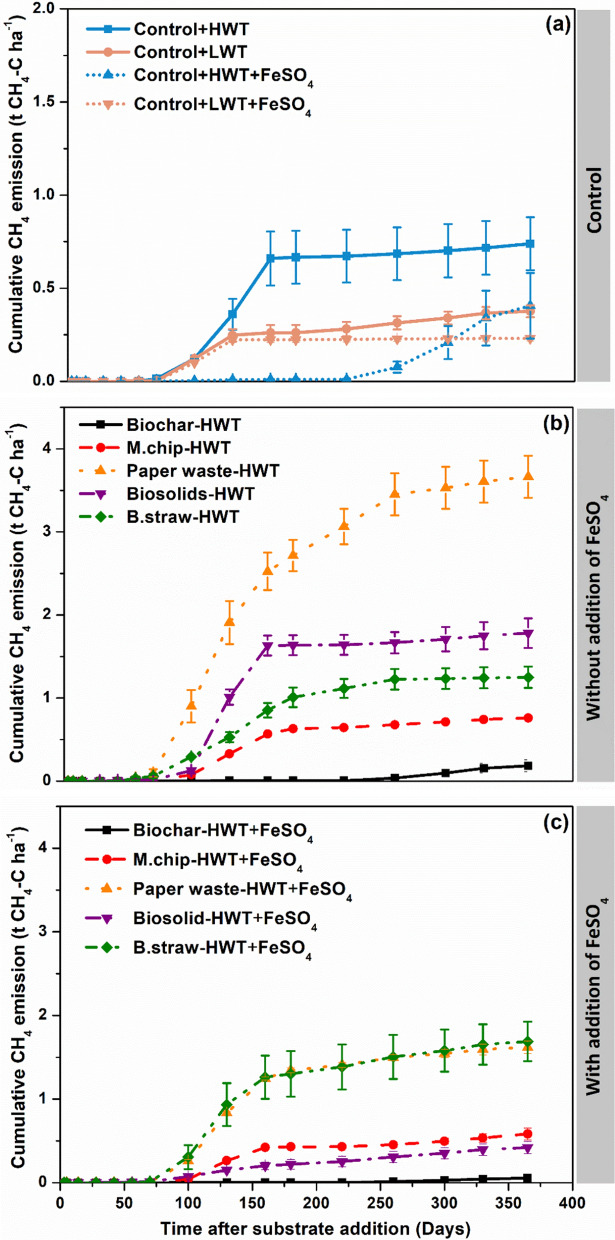
Effect of organic carbon amendments and FeSO_4_ addition on cumulative soil CH_4_ fluxes from an agricultural peat soil. The experiment had two controls consisting of a low water table (LWT) treatment and high-water table (HWT) without carbon amendments with and without FeSO_4_ addition. The carbon amendments included *Miscanthus* biochar (Biochar), *Miscanthus* chips (M.chip), paper waste, biosolids and barley straw (B.straw). Values represent means ± standard errors (*n* = 4)

Raising the water table increased CH_4_ emissions, by around a factor of two for the Control-HWT treatment compared to Control-LWT treatment (*P* = 0.005) (Fig. 2a). The largest cumulative CH_4_ emissions were observed in the Paperwaste-HWT treatment, however, this reduced 2.2-fold by the addition of FeSO_4_ (*P* = 0.003) (Fig. 2). CH_4_ emissions in the Biochar-HWT treatment (0.18 t CH_4_ ha^−1^ yr^−1^) were four times lower than in the Control-HWT treatment (0.73 CH_4_ ha^−1^ yr^−1^) (*P* = 0.021) (Fig. 2).

**Fig. 3:**
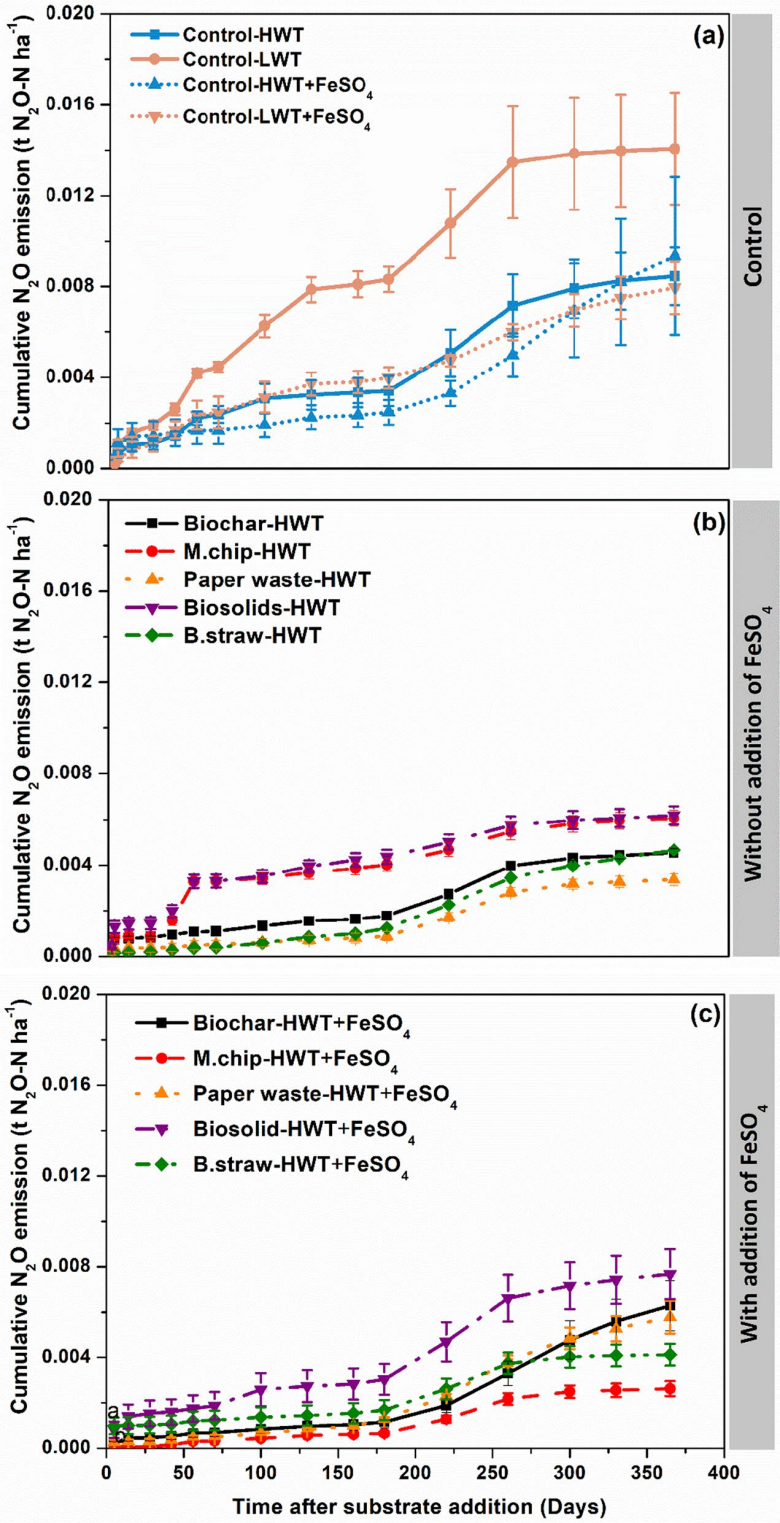
Effect of organic carbon amendments and FeSO_4_ addition on cumulative soil N_2_O fluxes from an agricultural peat soil. The experiment had two controls consisting of a low water table (LWT) treatment and high-water table (HWT) without carbon amendment with and without FeSO_4_ addition. The carbon amendments included *Miscanthus* biochar (Biochar), *Miscanthus* chips (M.chip), paper waste, biosolids and barley straw (B.straw). Values represent means ± standard errors (*n* = 4)

Cumulative N_2_O emissions were greater in the low water table treatments in the absence of FeSO_4_ (0.092 g N_2_O m^−2^ yr^−1^), however, raising the water table significantly reduced N_2_O emissions (0.055 g N_2_O m^−2^ yr^−1^) (P = 0.046) (Fig. 3). Organic amendments or FeSO_4_ addition to peat soils had a consistent impact on N_2_O emissions (Fig. 3b, c). Similarly, the addition of FeSO_4_ also reduced cumulative N_2_O emissions.

### Carbon storage potential

**Fig. 4:**
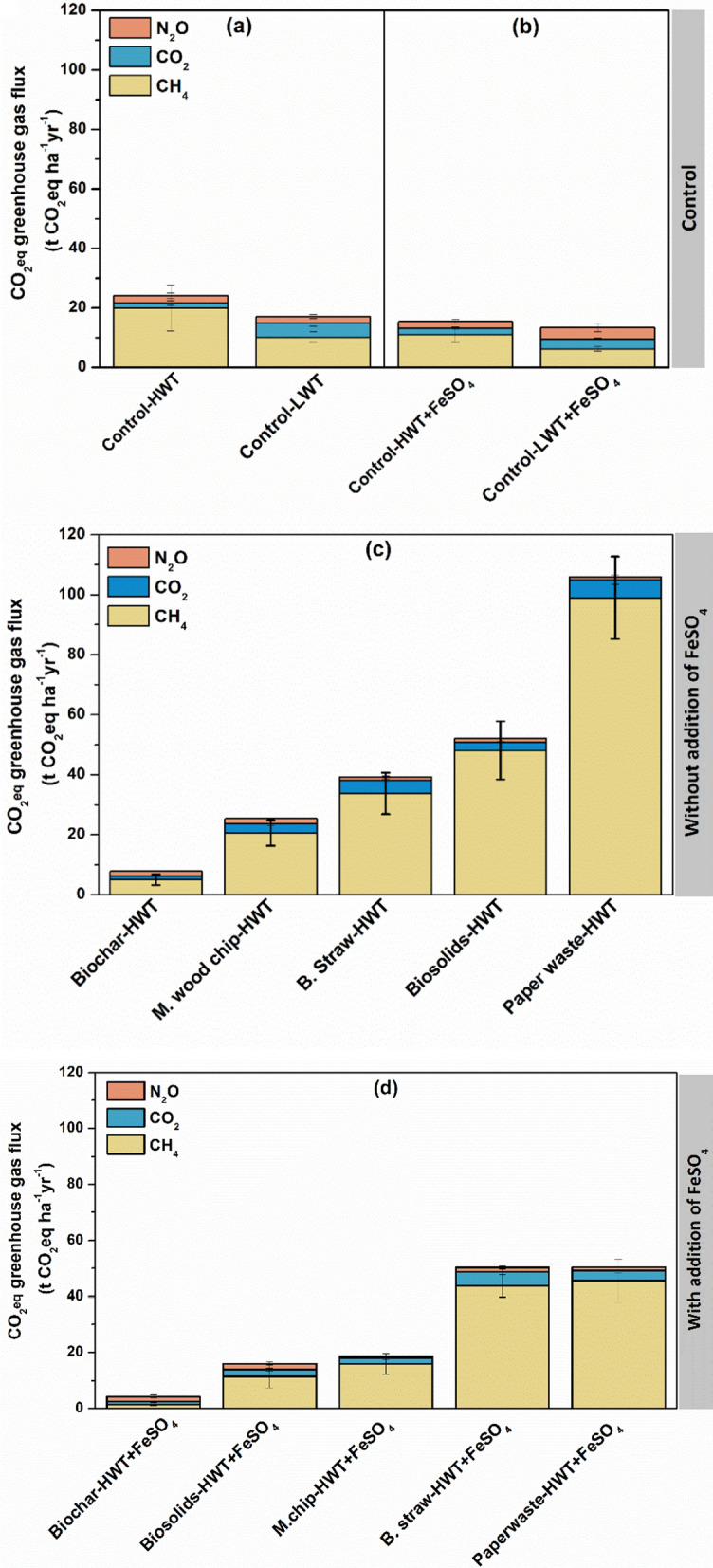
Effect of organic carbon amendment and FeSO_4_ addition on greenhouse gas emissions when expressed in CO_2_ equivalents (panels a b and c). GWP was based on radiative forcing over a 100-years’ time horizon: CO_2_ = 1, CH_4_ = 28, and N_2_O = 265. The carbon amendments included *Miscanthus* biochar (Biochar), *Miscanthus* chips (M.chip), paper waste, biosolids and barley straw (B.straw). The experiment had two control consisting of a low water table (LWT) treatment and high water table (HWT) without carbon amendement with and without FeSO_4_ addition. Values represent mean ± standard errors (*n* = 4)

The impacts of soil amendments and water table on C storage aligned with the observed patterns in GHG dynamics. After 1 year, soil C loss was the lowest with biochar addition (2.42–3.15 t C ha^−1^ yr^−1^), with and without FeSO_4_ addition, respectively, but markedly higher in the paper waste and biosolids treatments (15.2 and 22.1 t C ha^−1^ yr^−1^, respectively) (*P* = 0.0003) (Fig. S4). In the control treatments, total C storage after 1 year was 160.8–166.9 t C ha^−1^ in the low and high-water table treatments, indicating net soil C losses of 11.6–5.5 t C ha^−1^ soil over the experiment duration (*P* = 0.013) (Fig. 4a).

### Net GHG emissions

When expressed as CO_2_ equivalents (CO_2_eq) over a 100-year time frame, cumulative annual GHG emissions varied greatly among controls, ranging from 6.6 to 11 and 10 to 20 t CO_2_eq ha^−1^ yr^−1^ in the presence and absence of added FeSO_4_, respectively (Fig. 4a). Overall, the addition of FeSO_4_ substantially reduced the total GHG balance (*P* = 0.07). Under high water table conditions, biochar treatments significantly decreased net GHG emissions relative to the controls, with or without added FeSO_4_ (*P* = 0.016)_._ In contrast, Paper waste amendment markedly increased emissions of CO_2_, CH_4_ and overall net GHG release compared to the unamended controls (*P* = 0.0001). Across all high-water table treatments, CH_4_ comprised > 50% of total net GHG balance, followed by CO_2_, while N_2_O made a negligible contribution (< 0.5%).

## Discussion

### CO_2_ emissions

Cumulative CO_2_ emissions in the low water table (Control-LWT) was 2.9 times higher than its counterpart with a high-water table (Control-HWT) throughout the 365-day experimental period (*P* = 0.0053) (Fig. 1a, b, Table 1). This finding aligns with previous studies showing lower rates of peat oxidation and resulting CO_2_ emissions under anaerobic high water table conditions (Knox et al. 2015). In contrast, raising the water table (similar to Control-HWT) is generally considered an effective way to reduce C loss in cultivated peatlands, as it decreases soil aeration and microbial peat mineralization (Wen et al. 2020; Evans et al 2021). However, the continued (albeit reduced) emissions from the HWT treatment (1.68 ± 0.35 t CO_2_ ha^−1^ yr^−1^) are likely due to the absence of C input to the cores, which were unvegetated, and some degree of oxygen ingress to the exposed peat surface (Boonman et al. 2022).

Cumulative CO_2_ emissions in the organic amendments ranged from 1.2 to 6.04 t CO_2_ ha^−1^ yr^−1^ with the lowest emission observed in the Biochar-HWT treatment and highest in the Paper waste-HWT treatment (*P* = 0.008) (Fig. 1a). Biochar addition resulted the lowest CO_2_ emission among all the treatments, suppressing emissions by 30% compared to the unamended Control-LWT (*P* = 0.005), consistent with studies in different soil systems (Wardle et al. 2008; Spokas et al. 2012; Spokas 2013). These results support the growing body of evidence that biochar, in addition to being a recalcitrant form of C in the soil, may also inhibit turnover of SOC (Woolf and Lehmann 2012; Jeffery et al. 2015). Furthermore, the suppression of soil CO_2_ emissions may be attributed to the high C/N ratio (> 100) of biochar, which reduces the mineralization intensity and weakens enzymatic activity. Additionally, the precipitation of CO_2_ onto the biochar surface suppressing the CO_2_ emission (Case et al. 2014). Overall, our data indicate that this inhibition process, commonly studied in mineral soils, also occurs in peatlands. This finding supports our initial hypothesis that adding high C:N ratio amendments under high water table conditions would promote C storage.

### CH_4_ emissions

Raising the water table increased CH_4_ emissions, by around a factor of two for the Control-HWT treatment compared to Control-LWT treatment (P = 0.005) (Fig. 2a). This is consistent with a reduction in oxygen ingress to the waterlogged soil producing anaerobic conditions, favoring methanogenic microbes (Thauer 1998; Gao et al. 2018). The differences in CH_4_ fluxes seem to be largely driven by a pulse of activity (days 100–175), corresponding to the preferred redox potential ranges for active methanogens were below − 100 mV (Liu et al. 2020b). The addition of the more labile C substrates (straw, biosolids and paper waste) to HWT cores led to a further, large increases in CH_4_. We ascribe this response to the greater availability of labile organic compounds, which are fermented into acetate, a methanogenic substrate (Chandra et al. 2012; Christy et al. 2014; Chojnacka et al. 2015).

In marked contrast, CH_4_ emissions in the Biochar-HWT treatment were four times lower than those in the Control-HWT treatment (*P* = 0.0004) (Fig. 2b), suggesting a strong suppressive effect from biochar addition. This is consistent with a previous study of *Miscanthus* biochar application to mineral soil (Case et al. 2014) and with other studies in biochar-amended peat soils (Davidson et al. 2019; Sun et al. 2021). Potential mechanisms for the strong suppressive effect of biochar on CH_4_ emissions include: (i) altering the soil redox environment and accelerating electron transfer, which facilitates organic matter oxidation through non-methanogenic pathways; (ii) immobilizing labile organic substrates; (iii) providing a matrix and aerobic microsites for methanotrophic bacteria, promoting CH_4_ oxidation (Lovley et al. 2004; Jeffery et al. 2016).

The addition of FeSO_4_ had a generally negative impact on CH_4_ fluxes, reducing emissions by 39% for the Control-LWT treatment, and 45% for the Control-HWT treatment (Fig. 2c). Addition of FeSO_4_ also led to reductions in CH_4_ emissions for the paper waste, biosolids, *Miscanthus* chip and biochar amendments (relative to the corresponding treatment without FeSO_4_) of between 54% and 72%. The only exception was for the straw application, where CH_4_ emissions were 35% higher from the FeSO_4_-amended cores, but very high (> 1250 kg CH_4_ ha^−1^ yr^−1^) in both treatments. The generally suppressive effect of FeSO_4_ on CH_4_ emissions in the other treatments is consistent with the our last hypothesis that the presence of SO_4_^2−^ (and potentially also Fe^3+^) in the pore waters of agricultural peat soils can inhibit methanogenesis by providing alternative electron acceptors for organic matter oxidation, which outcompete methanogens (Gauci et al. 2004; Blodau et al. 2007; Pester et al. 2012).

### N_2_O emissions

Raising the water table reduced N_2_O emissions in comparison to the Control-LWT treatment (Fig. 3a, b). In natural peatlands, N_2_O emissions are generally low due to the little availability of oxygen and/or nitrogen (Klemedtsson et al. 2005). Drainage and fertilization of agricultural peatlands increase N_2_O emissions by enhancing oxygen and mineral nitrogen availability (Klemedtsson et al. 2005; Pärn et al. 2018). Confirming our third hypothesis, our cumulative N_2_O emission results suggest that re-wetting is an effective strategy to reduce N_2_O emissions from agricultural peatlands. On the other hand, we found little evidence that either organic amendments or FeSO_4_ addition to peat soils had a consistent impact on N_2_O emissions (Fig. 3b, c). The average N_2_O emission over the year were between 3 and 8.5 kg N_2_O-N ha^−1^. These results are similar to the IPCC Tier 1 N_2_O emission factor for temperate peat under cropland of 8.2 kg N_2_O-N ha^−1^ yr^−1^ (Liu et al. 2020a).

### Carbon balance and CO_2_ equivalent greenhouse gas emission

**Fig. 5:**
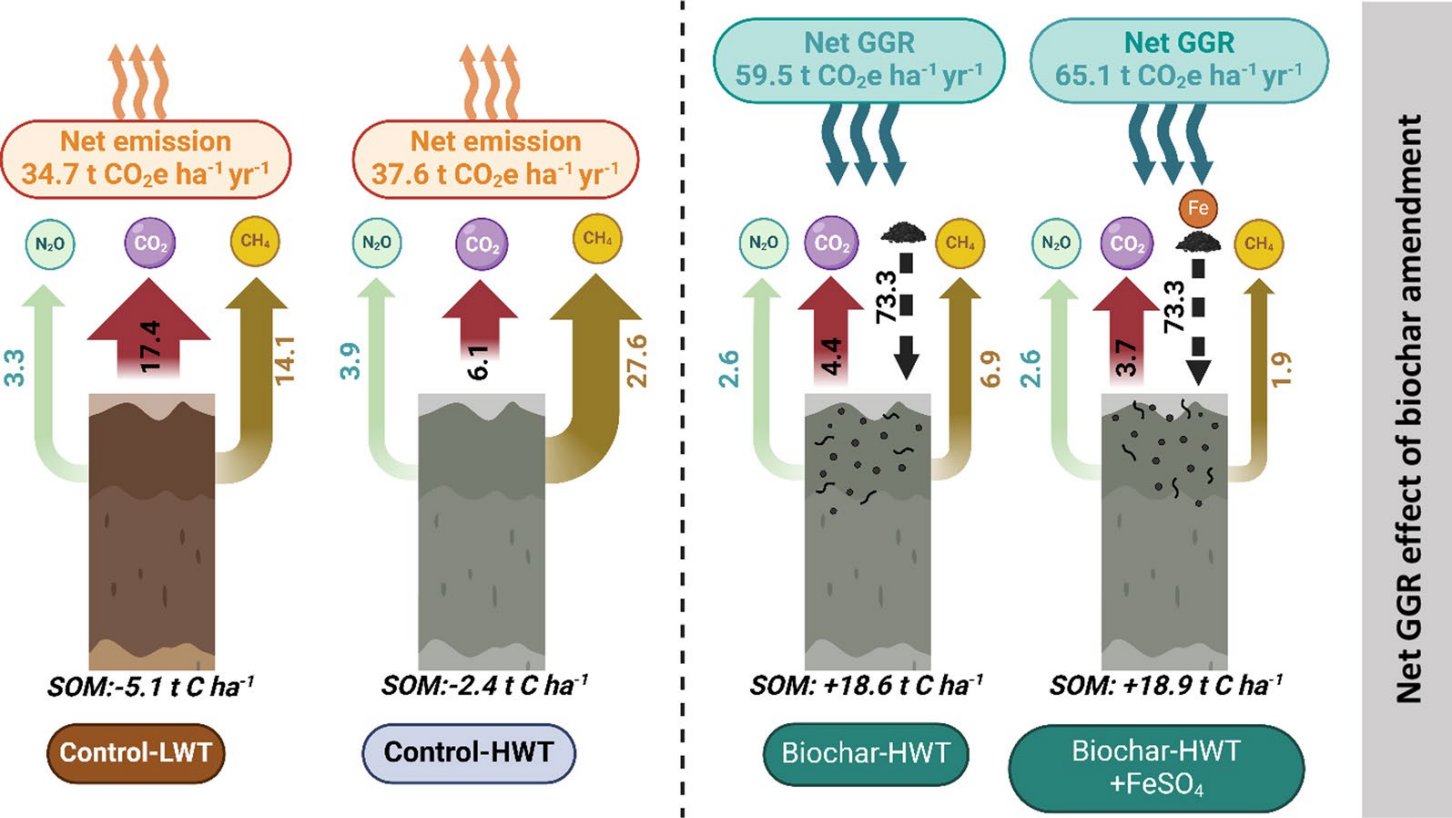
Concept diagram of estimated greenhouse gas and soil carbon balance for peat mesocosms with biochar amendment (Biochar-HWT and Biochar-HWT + FeSO_4_) treatments, compared to control mesocosms under two different water table levels (Control-LWT and Control-HWT)

In the Control-LWT treatment, which received no organic and inorganic amendments, cumulative CO_2_ plus CH_4_ emissions equated to a soil C loss of 5.1 t C ha^−1^ yr^−1^ within 1 year, representing approximately 3% of the original C stock of the peat core (Fig. 5). For the Control-HWT treatment, this was reduced to 2.4 t C ha^−1^ yr^−1^ (1.4% of the initial C stock) (*P* = 0.013). For all treatments amended by organic matter, measured C losses were < the 20 t C ha^−1^ of C added, indicating that they acted as net C sinks. However, for the more labile organic matter amendments (paper waste, straw, and biosolids), measured C losses accounted for between 25% and 50% of the added C, suggesting that these treatments would be unlikely to remain net C sinks over a longer time period after the initial application.

In contrast, C losses from the *Miscanthus* chip treatment represented 20% of inputs, and for biochar they were only 7%, suggesting that these more recalcitrant amendments would more likely result in long-term C sequestration. Addition of FeSO_4_ further reduced C losses to 14 and 5% of input for *Miscanthus* chip and biochar respectively.

Based on 100-year global warming potentials for CH_4_ and N_2_O, overall GHG emissions were similar for the Control-LWT and Control-HWT treatments (34.7 and 37.6 t CO_2_eq ha^−1^ yr^−1^ respectively) due to counterbalancing lower in CO_2_ and higher CH_4_ emissions from the HWT cores. Our results imply that simple re-wetting would not result in net GHG emission reductions at this site, at least in the first year of raised water levels. In the longer term, however, the radiative forcing benefits of conserving peatland C stocks via re-wetting can be expected to outweigh the costs of higher CH_4_ emissions due to the shorter atmospheric lifetime of CH_4_ (Günther et al. 2020).

The GHG balance of organic matter amended cores (considering the organic matter input as CO_2_ sequestration) varied greatly, from −59.5 t CO_2_eq ha^−1^ yr^−1^ for the biochar-HWT treatment (i.e. adding biochar resulted in a strong net GHG sink) to + 87.1 t CO_2_eq ha^−1^ yr^−1^ for the Paper waste-HWT treatment (*P* = 0.0009) (Table 1; Fig. S2). This positive high emission was the result of very high CH_4_ emissions. All other organic amendments had lower GHG emissions relative to the controls, but apart from biochar the only other amendment that resulted in net GHG removal over one year was *Miscanthus* chip (Fig. S2). Again, high CH_4_ emissions from the treatments with more labile organic amendments strongly offset the CO_2_ sequestration (Table 1). In general, N_2_O made only a minor contribution to overall GHG emissions.

Adding FeSO_4_ had a net negative effect on the GHG balance in both LWT and HWT controls, and for all organic amendments other than straw (Fig. 5). This resulted from suppression of both CO_2_ and CH_4_ emissions. The strongest measured GHG removal was for the Biochar-HWT + FeSO_4_ treatment (−65.1 t CO_2_eq ha^−1^ yr^−1^). Compared to the ‘business as usual’ Control-LWT counterfactual, the net climate mitigation benefit resulting from this treatment was 99.8 t CO_2_eq ha^−1^ yr^−1^ (*P* = 0.00004). These results compare highly favorably with estimates of negative emissions potential for other land-based interventions ranging 2.2–14.3 t CO_2_eq ha^−1^ yr^−1^ (Lee and Day 2012; Paustian et al. 2016; Alcalde et al. 2018).

### Implications for GHG removals

Our study suggests that the application of recalcitrant organic material to re-wetted agricultural peatlands could offer a highly effective and space-efficient climate change mitigation measure, delivering substantial GGR. Biochar application was found to be the most effective GGR option; in addition to low decomposition of the applied material, it suppressed both decomposition of the existing peat organic matter and CH_4_ emissions. Both suppressive effects were enhanced by the co-application of FeSO_4_. In comparison, re-wetting alone did not produce a net GHG emissions reduction due to increased CH_4_ emissions, and application of labile organic materials led to large additional increases in CH_4_, which largely negated any CO_2_ sequestration benefits. Although these CH_4_ emissions were in most cases also partly suppressed by FeSO_4_ co-application, we conclude that adding these forms of organic matter to wet peat soils is unlikely to deliver effective climate change mitigation.

We recognize that our findings are subject to several important caveats. Firstly, we only measured GHG fluxes for one year following application of the treatments (Harpenslager et al. 2024). Given that not all of the added organic matter was oxidized during this time, and that cumulative CO_2_ emissions continued to rise throughout the study period (Fig. 1), some further emissions would be expected, likely negating any remaining climate mitigation benefit of labile organic matter additions. On the other hand, CH_4_ emissions had slowed or even largely ceased, following an initial peak (Fig. 2), so the warming impact of CH_4_ (and the need to suppress emissions, e.g. via FeSO_4_ addition) may be largely limited to the first-year post-application. Even under the highly pessimistic assumptions that all CO_2_ emissions observed from the Biochar-HWT treatment were due to biochar oxidation, and that this oxidation rate remains constant for ten years, this treatment would still deliver strong GGR on this timescale (Case et al. 2014; Harpenslager et al., 2024). Given that CO_2_ emissions from this treatment were considerably lower than the Control-HWT treatment, it seems more likely that they were associated with peat oxidation, and that the biochar oxidation rate in this anaerobic environment was negligible.

A second caveat is that we did not undertake a full lifecycle assessment of the amendments added, and so our analysis does not take account of emissions associated with (for example) the production of biomass, pyrolysis of biochar, production of FeSO_4_, or transportation of materials (Xia et al. 2024). While these emissions may be partly mitigated where the amendments represent waste products or byproducts of industrial processes (e.g. biosolids, paper waste, and FeSO_4_), or where their production can also generate energy (e.g. pyrolysis), they will nevertheless reduce the overall effectiveness of these amendments as climate mitigation measures relative to our "site-level" calculations (Gauci and Chapman 2006). Equally, the economic feasibility of management practices like biochar addition or water table level manipulation remains uncertain and will depend on C permanence and C pricing. On the other hand, there is a recognized need to conserve remaining carbon stocks in agriculturally drained peat soils at a global level (Kasimir et al., 2018; Buschmann et al. 2020), and our analysis strongly suggests that the application of biochar (with or without FeSO_4_) would substantively shift the outcome of re-wetting towards lower peat CO_2_ and CH_4_ emissions, in addition to the direct carbon input associated with the biochar. On this basis, we conclude that biochar amendments to peatlands offer a potential "win–win" in terms of reductions in existing high GHG emissions, and new C capture and storage in a stable, anaerobic environment (Fig. 5). Concerted global efforts to apply biochar to re-wetted agricultural peatlands may therefore have the potential to make a significant contribution to achieving net zero targets. Such an approach would be consistent with recent policy and regulatory developments in Europe (Buschmann et al. 2020) and with the growing national, transnational, and private-sector investments in nature-based solutions for climate change mitigation (Workman et al. 2022). By overcoming the recognized trade-off between CO_2_ and CH_4_ emissions from wetland ecosystems, both biochar and FeSO_4_ application could enable peatlands make a significant contribution towards the diverse range of GGR options that are required to reach net zero.

## Conclusion

Here, we focused on GGR methods on agricultural peat lands using organic and inorganic amendments combined with water table manipulation. Our findings highlighted that, maintaining high water table level with FeSO_4_ were the main variables of significance. Amendments with a low C:N ratio, such as paper waste, straw, and biosolids, significantly increased GHG emissions from peat soils. This increase was due to the removal of N constraints associated with the labile C fraction and changes in the microbial community. However, biochar decreased GHG emissions. Raising the water table decreased CO_2_ emissions by inhibiting SOC decomposition, but increased CH_4_ emissions in the Paper waste treatment by methanogenesis. Biochar-HWT + FeSO_4_ strongly decreased the CO_2_ equivalents GHG emission. Overall, the combination of Biochar-HWT with FeSO_4_ presents a viable management strategy for mitigating GHG emissions in agricultural peatlands.

## Supplementary Information

Additional file 1.

## Data Availability

Data will be made available on request.
