# Supplementary material for: Greenhouse gas removal in agricultural peatland via raised water levels and soil amendment
Source: Biochar. 2025 Feb 21;7(1):39. doi: 10.1007/s42773-024-00422-2 (PMC11845426; doi:10.1007/s42773-024-00422-2)
Supplement: Supplementary file 1 — Additional file 1. [file 42773_2024_422_MOESM1_ESM.docx]

**Greenhouse gas removal in agricultural peatland via raised water levels and soil amendment**

Peduruhewa H. Jeewani^a,*^, Robert W. Brown^a^, Jennifer M. Rhymes^b^, Niall P. McNamara^c^, David R. Chadwick^a^, Davey L. Jones^a^, Chris D. Evans^b^

^a^ *School of Environmental and Natural Sciences, Bangor University, Bangor, Gwynedd, LL57 2UW, UK*

^b^ *UK Centre for Ecology and Hydrology, Bangor, Gwynedd, LL57 2UW, UK*

^c^ *UK Centre for Ecology & Hydrology, Lancaster, LA1 4AP, UK*

^*^Corresponding author: Peduruhewa H. Jeewani

Corresponding Author Address: School of Environmental and Natural Sciences, Bangor University, Bangor, Gwynedd, LL57 2UW, UK

Corresponding Author Email: j.hemamali@bangor.ac.uk

**Table S1.** Physicochemical properties of soil and applied amendments used in the experiments. Values represent means ± standard errors (n = 4). Abbreviation: C, carbon; N, nitrogen; SOM, Soil organic matter content; EC, electrical conductivity; BD, bulk density. Where applicable, the data is expressed on a dry weight basis.

| Properties | Soil | | | *Miscanthus*  biochar | *Miscanthus* chip | Paper  waste | Barley  straw | Biosolids |
| --- | --- | --- | --- | --- | --- | --- | --- | --- |
|  | 0-20 cm | 10-30 cm | 30-50 cm |  |  |  |  |  |
| Total C (%) | 27.6±2.6 | 27.6±1.7 | 24.7±0.9 | 79.3±0.9 | 47.4±0.5 | 46.8±0.81 | 43.7±0.5 | 36.45±30 |
| Total N (%) | 1.81±0.45 | 1.81±0.13 | 1.80±0.08 | 0.41±0.01 | 0.49±0.07 | 0.75±0.07 | 0.68±0.02 | 3.66±6 |
| C:N ratio | 16.0±4.8 | 15.2±1.3 | 13.7±0.9 | 259±14 | 97.9±16 | 62.8±7.3 | 63.5±2.0 | 9.95±0.03 |
| SOM (%) | 45.22±2.6 | 33.05±5.6 | 20.79±1.9 | - | - | - | - | - |
| pH (H_2_O) | 6.54±0.05 | 6.65±0.04 | 6.54±0.04 | 5.6± 0.61 | 6.9±0.85 | 5.19±0.52 | - |  |
| EC (μS cm^−1^) | 193±4 | 186±4 | 199±9 | - | - | - | - | - |
| BD (g cm^-3^) | 0.52±0.05 | 0.55±0.03 | 0.53±0.03 | - | - | - | - | - |
| NO_3_^-^ (mg N L^-1^) | 4.05±0.29 | 3.52±0.36 | 3.54±0.29 | 0.15±0.01 | 0.11±0.02 | 0.3±0.03 | 5.84±0.98 | 1.7 ±0.2 |
| NH_4_^+^ (mg N L^-1^) | 4.48±0.22 | 2.68±0.44 | 4.65±0.58 | 0.25±0.08 | 2.87±0.56 | 1.02±0.08 | 3.79±1.02 | 218±0.15.6 |
| SO_4_^2-^ (mg S L^-1^) | 1.35±0.42 | 1.29±0.34 | 1.40±0.30 | - | - | - | - | - |
| PO_4_^3-^ (mg P L^-1^) | 1.05±0.11 | 1.23±0.34 | 0.98±0.19 | 3.33±0.98 | 2.26±0.8 | 3.57±0.9 | 4.03±0.89 | 2.32±0.23 |

**Table S2.** Characteristics of biochar. The biochar assessment for atomic H/C ratio and the fraction of stable polyaromatic carbon (SPAC) determined by hydropyrolysis (Hypy test)

| Sample number | Carbon (%) | Hydrogen (%) | Nitrogen (%) | Atomic H/C | SPAC (%) | EC (µS cm^-1^) | pH |
| --- | --- | --- | --- | --- | --- | --- | --- |
| Sample 1 | 73.57 | 4.08 | 0.44 | 0.66 | 25.18 | 51.53 | 5.65 |
| Sample 2 | 72.7 | 4.27 | 0.39 | 0.69 | 22.08 | 52.01 | 6.21 |
| Sample 3 | 73.66 | 3.86 | 0.22 | 0.62 | 24.21 | 50.13 | 5.85 |


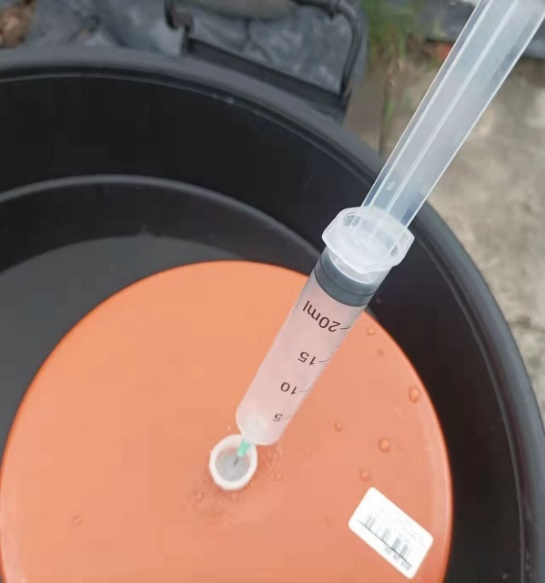

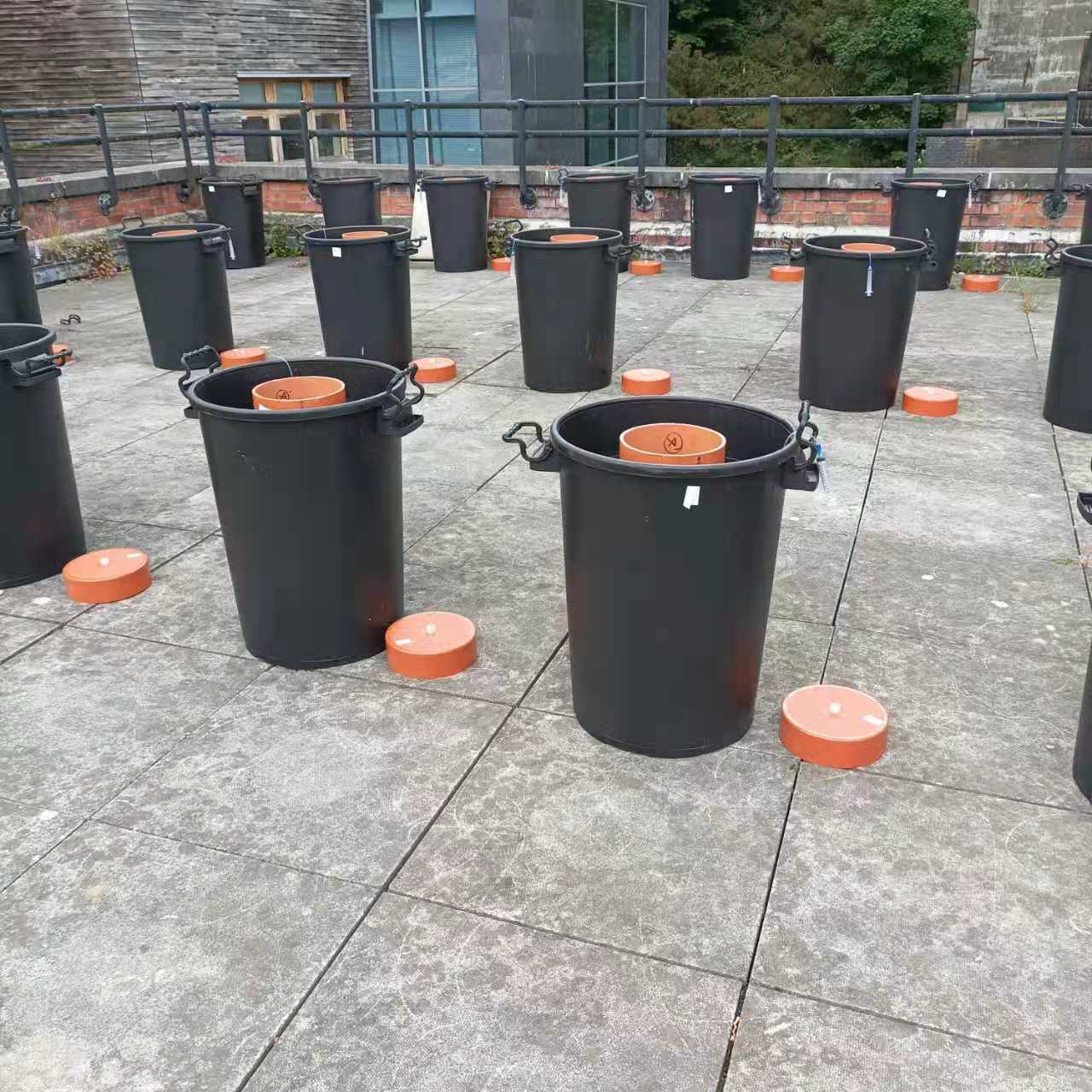


**B**

**A**

**A**

**Fig. S1:** Peat soil mesocosm setup. Intact peat soil mesocosm was placed into an outer container with drainage holes drilled to maintain the high or low water table level (A); headspace greenhouse gas sampling from the mesocosms after fitting a gas-tight lid (B).

**Net GGR effect of miscanthus chip amendment**


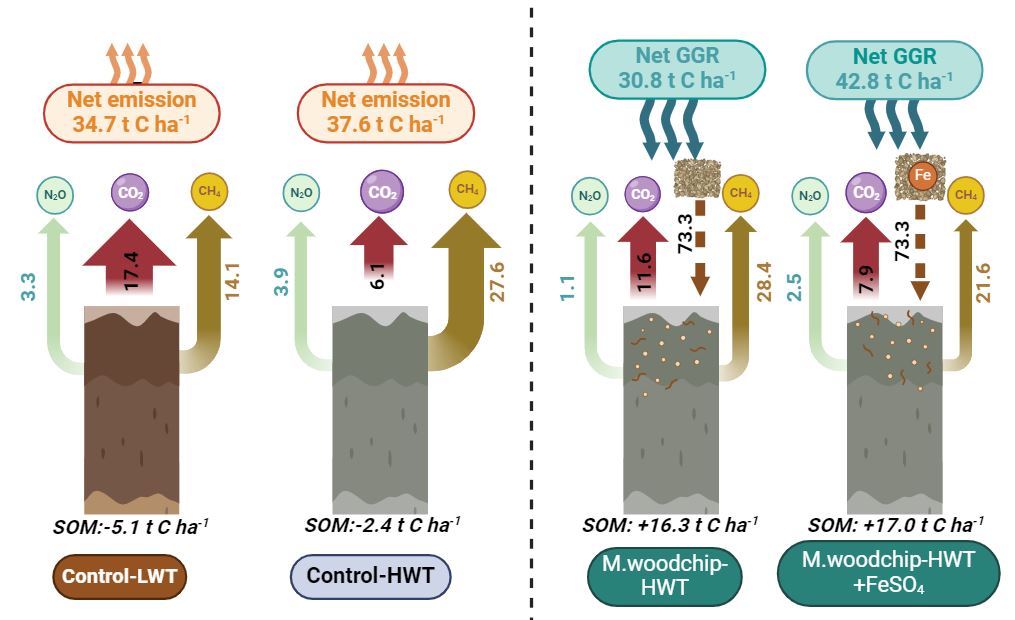

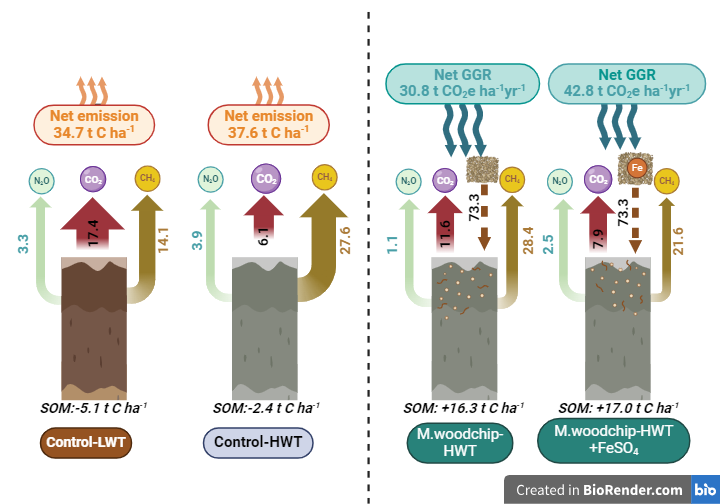

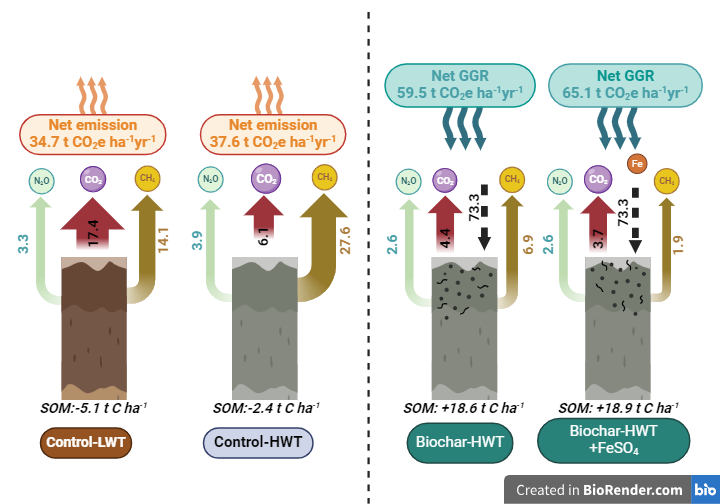

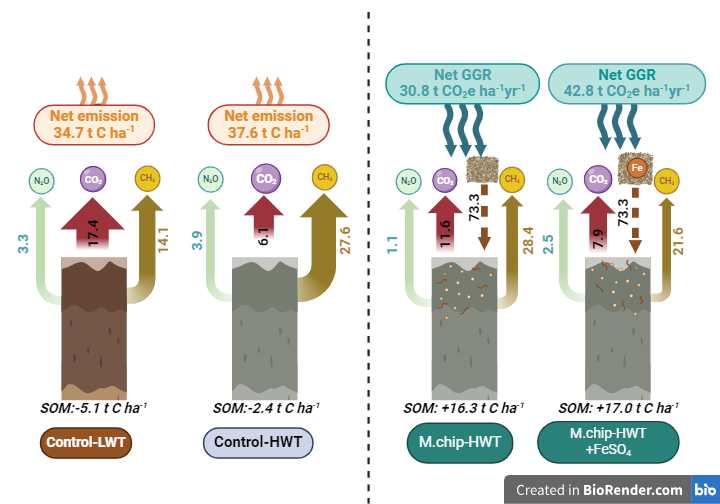


**Fig. S2:** Estimated greenhouse gas and soil carbon balance for peat mesocosms (M.chip-HWT and M.chip-HWT+FeSO_4_) treatments, compared to control mesocosms under two different water table levels (Control-LWT and Control-HWT). The C amendments included *Miscanthus* chips (M.chip). The experiment had two control consisting of a low water table (LWT) treatment and high water table (HWT) without C amendement with and without FeSO_4_ addition. Values represent mean ± standard errors (*n* = 4).

(b)

(a)


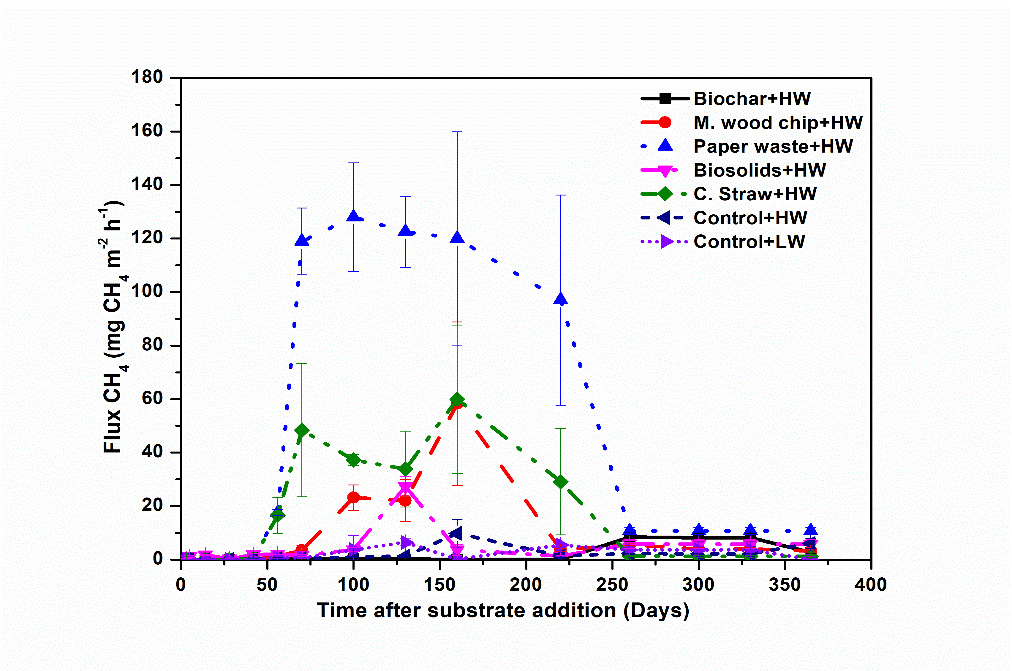

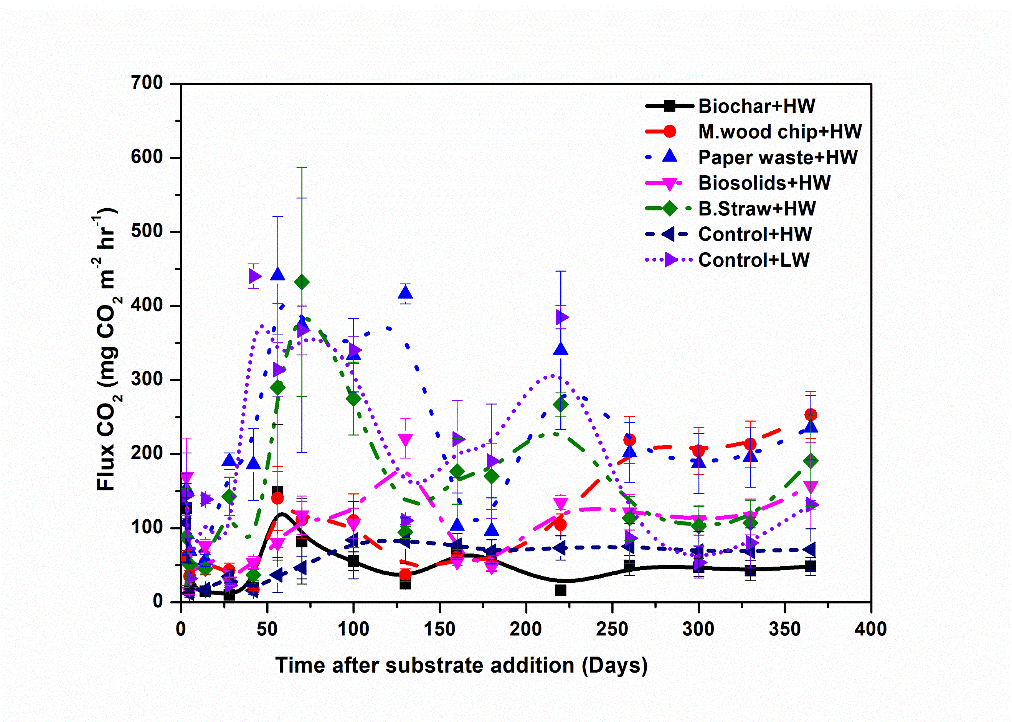

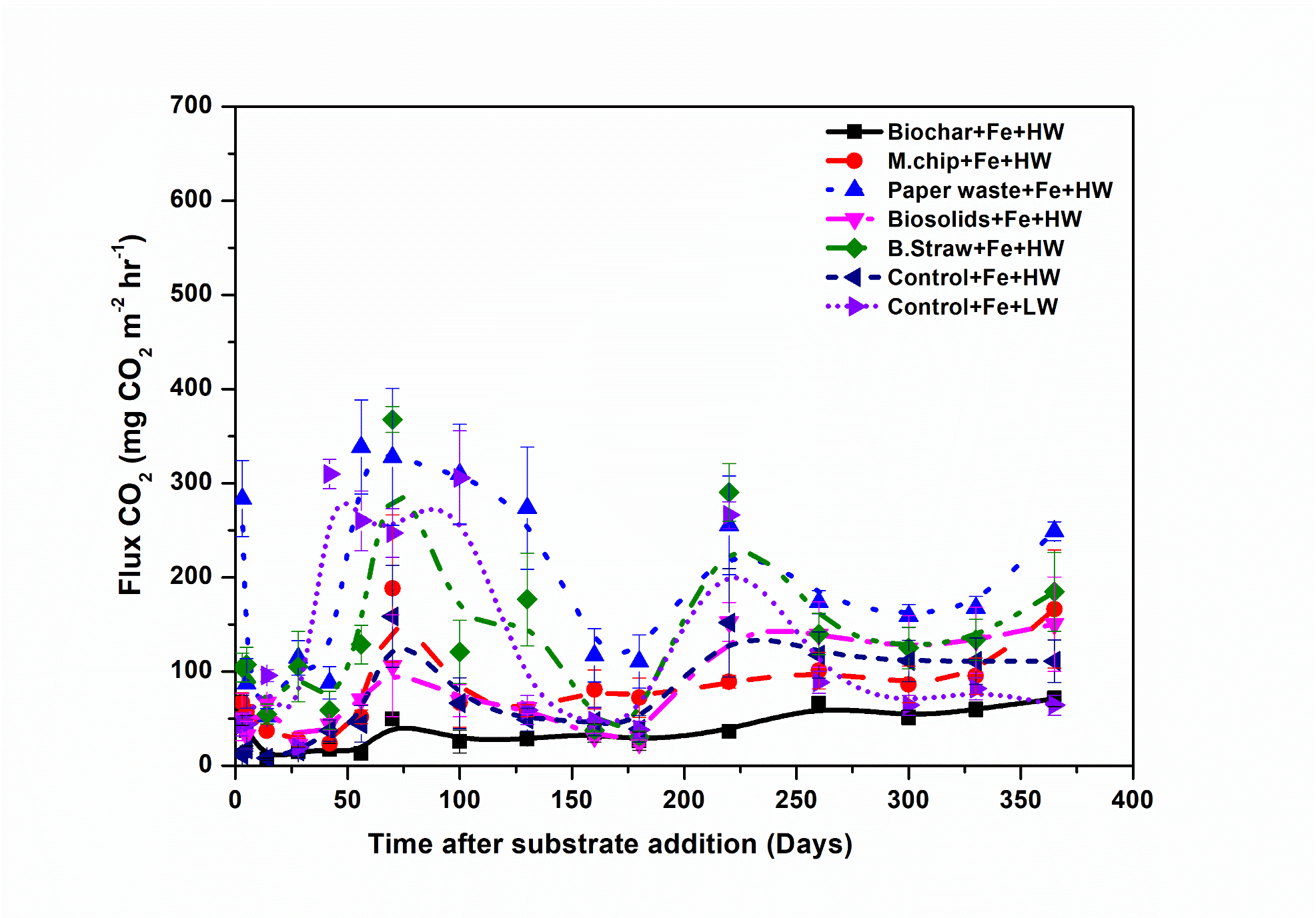


(c)

(d)





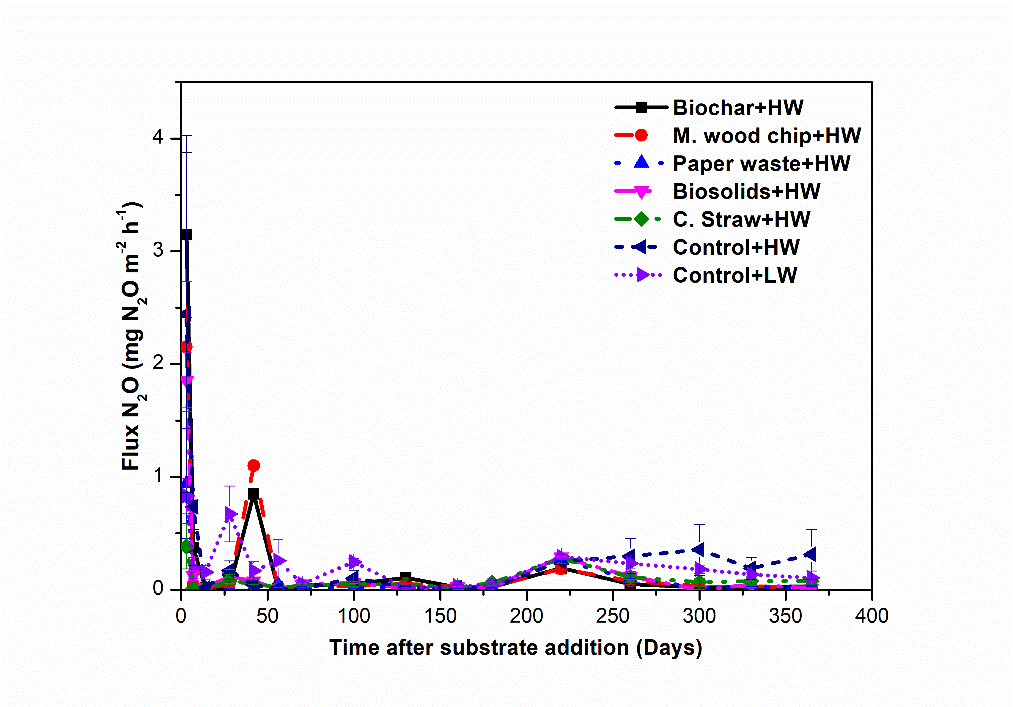


(e)

(f)


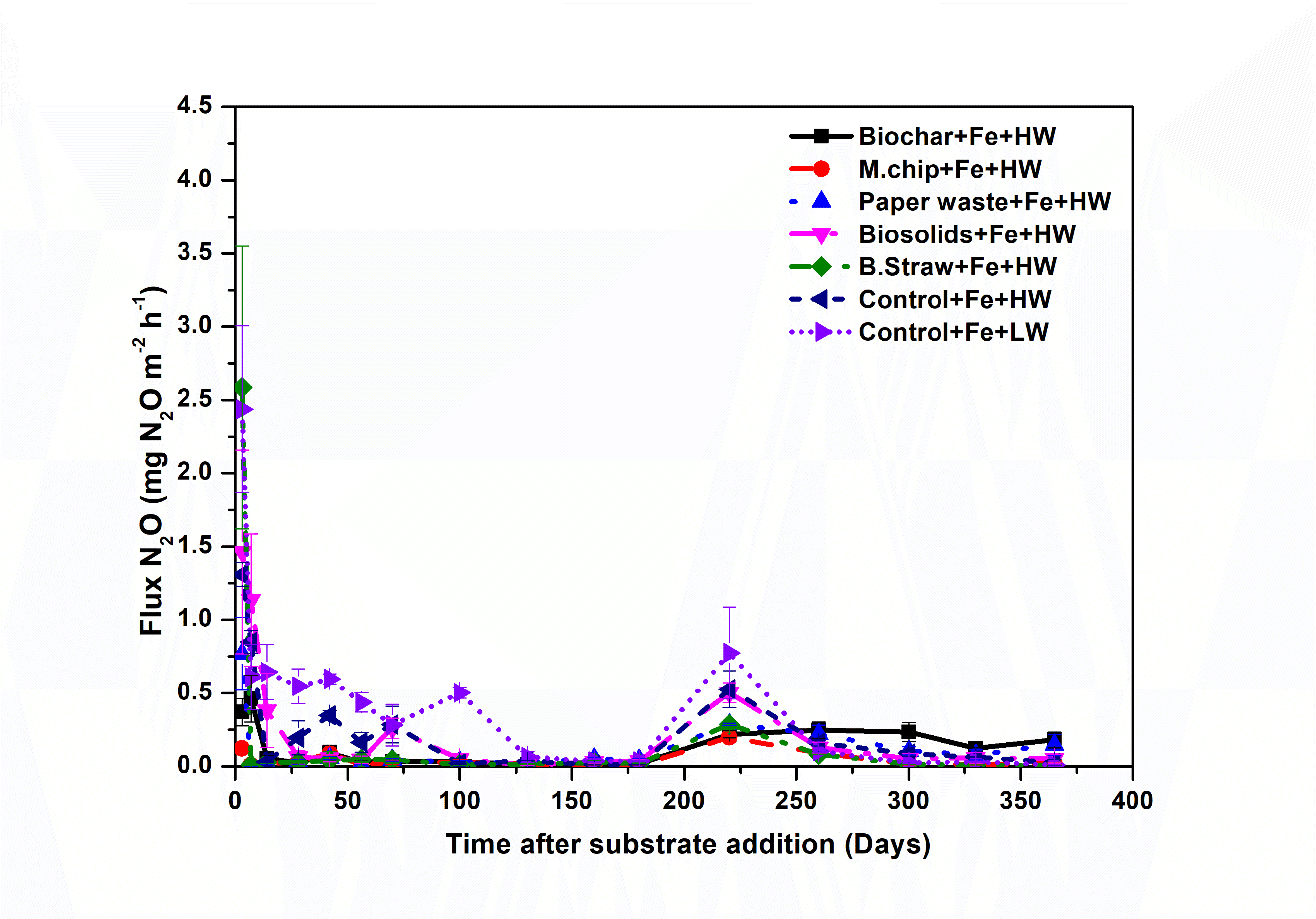


**Fig. S3*.*** Effect of organic C amendments and FeSO_4_ addition on daily soil CO_2_ (a,b)_,_ CH_4_ (c,d) and N_2_O (e,f) fluxes from an agricultural peat soil under a high (0 cm) water table. The C amendments included *Miscanthus* biochar (Biochar), *Miscanthus* chips (M.chip), paper waste, biosolids and barley straw (B.straw). The experiment had two controls consisting of a low water table (LWT) treatment and high-water table (HWT) without C amendment with and without FeSO_4_ addition. Values represent means ± standard errors (*n* = 4).


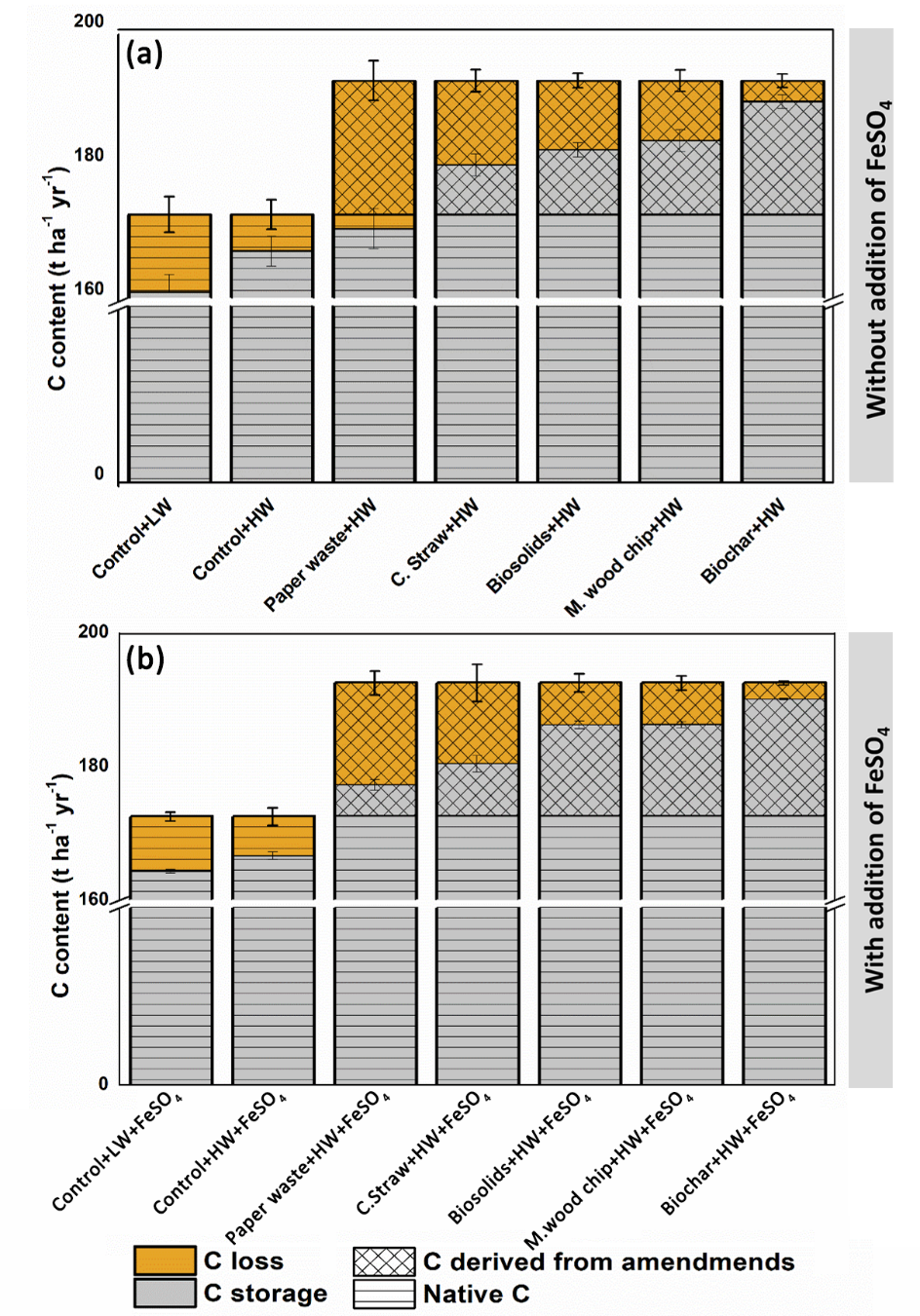

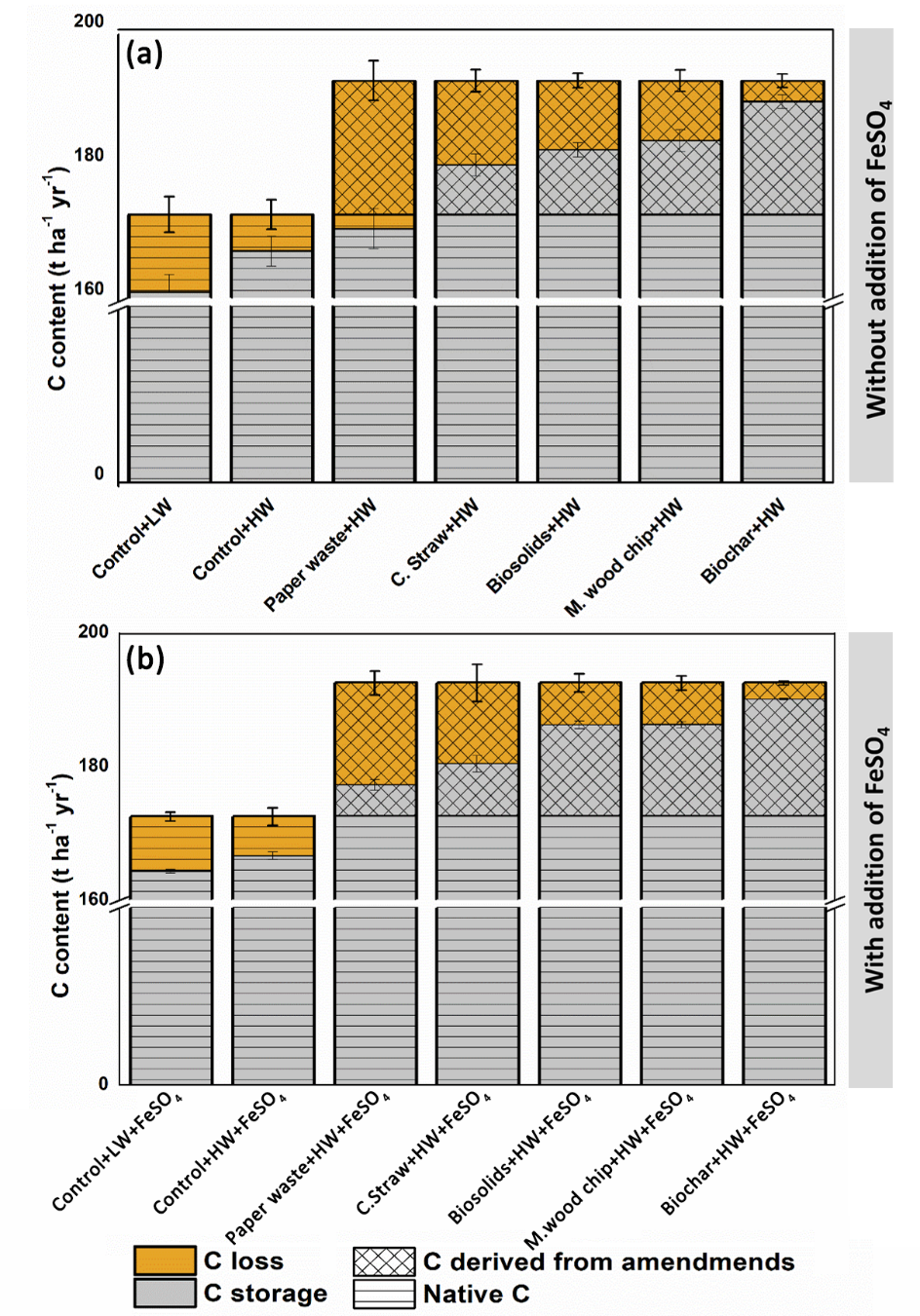

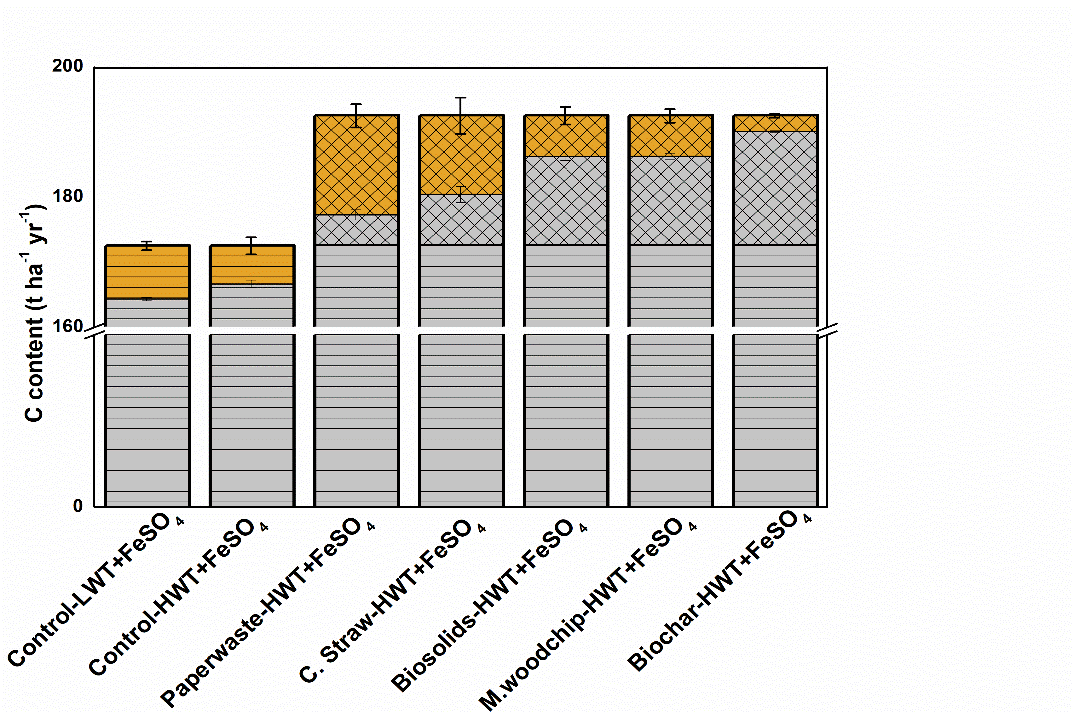

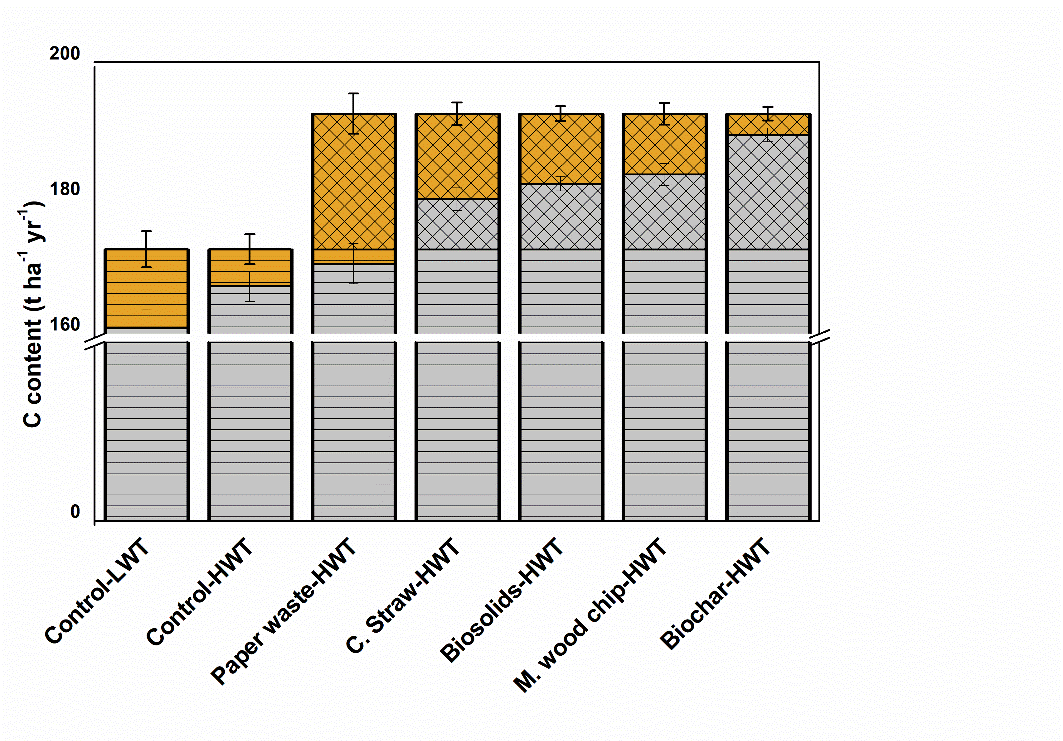

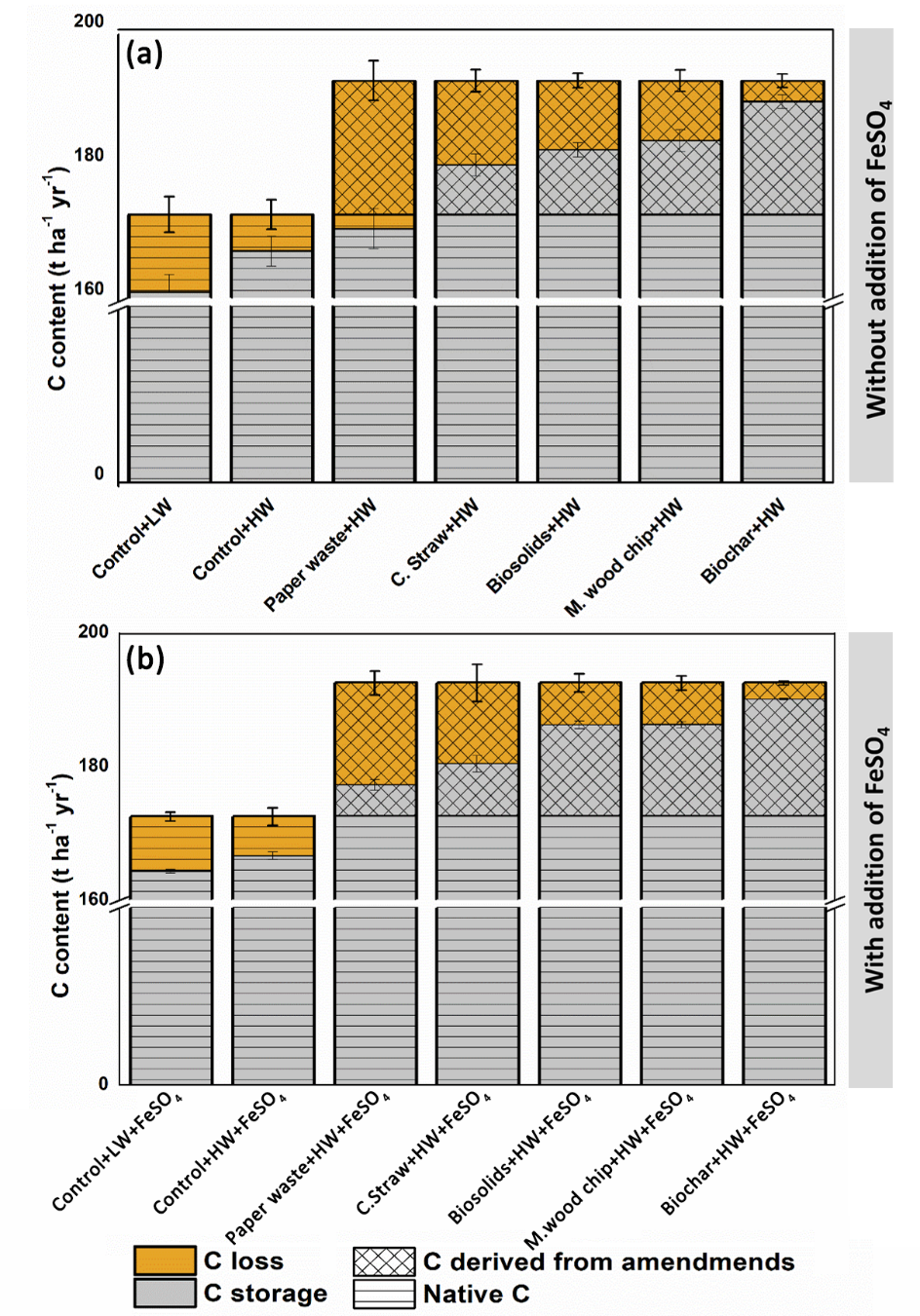

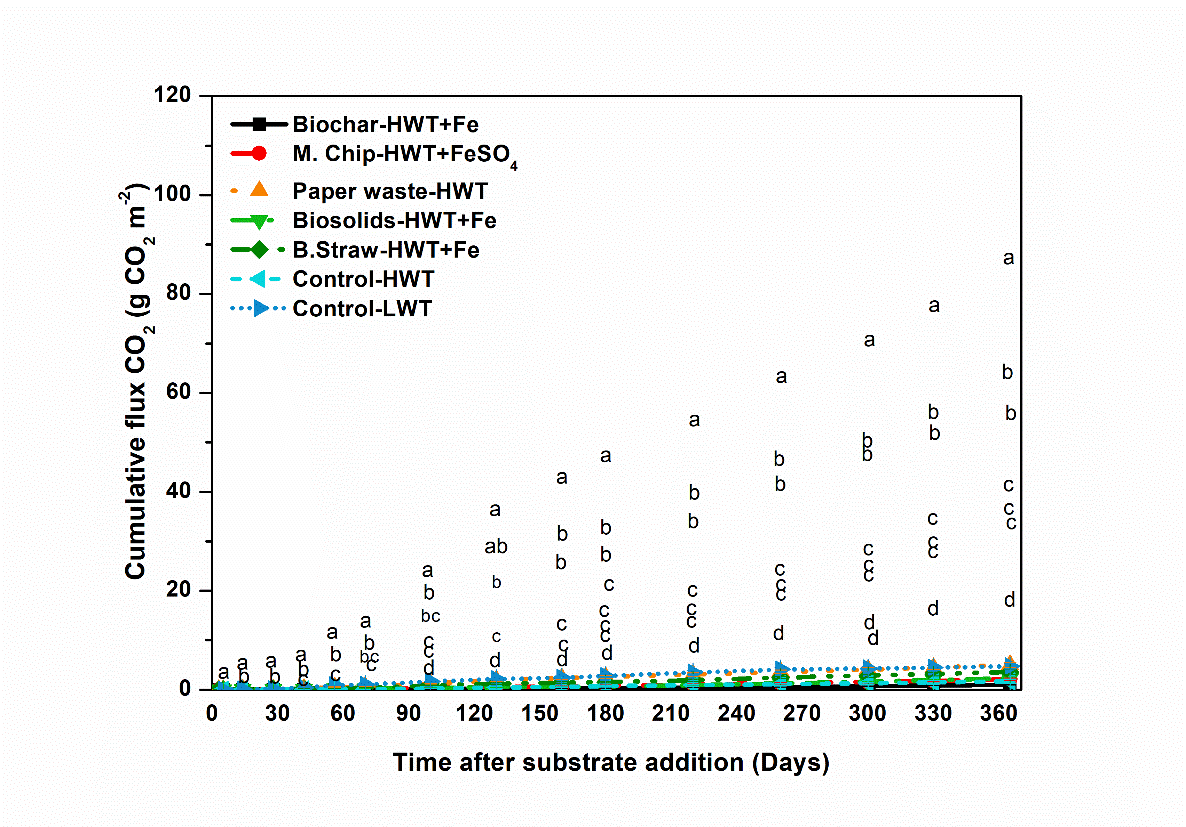

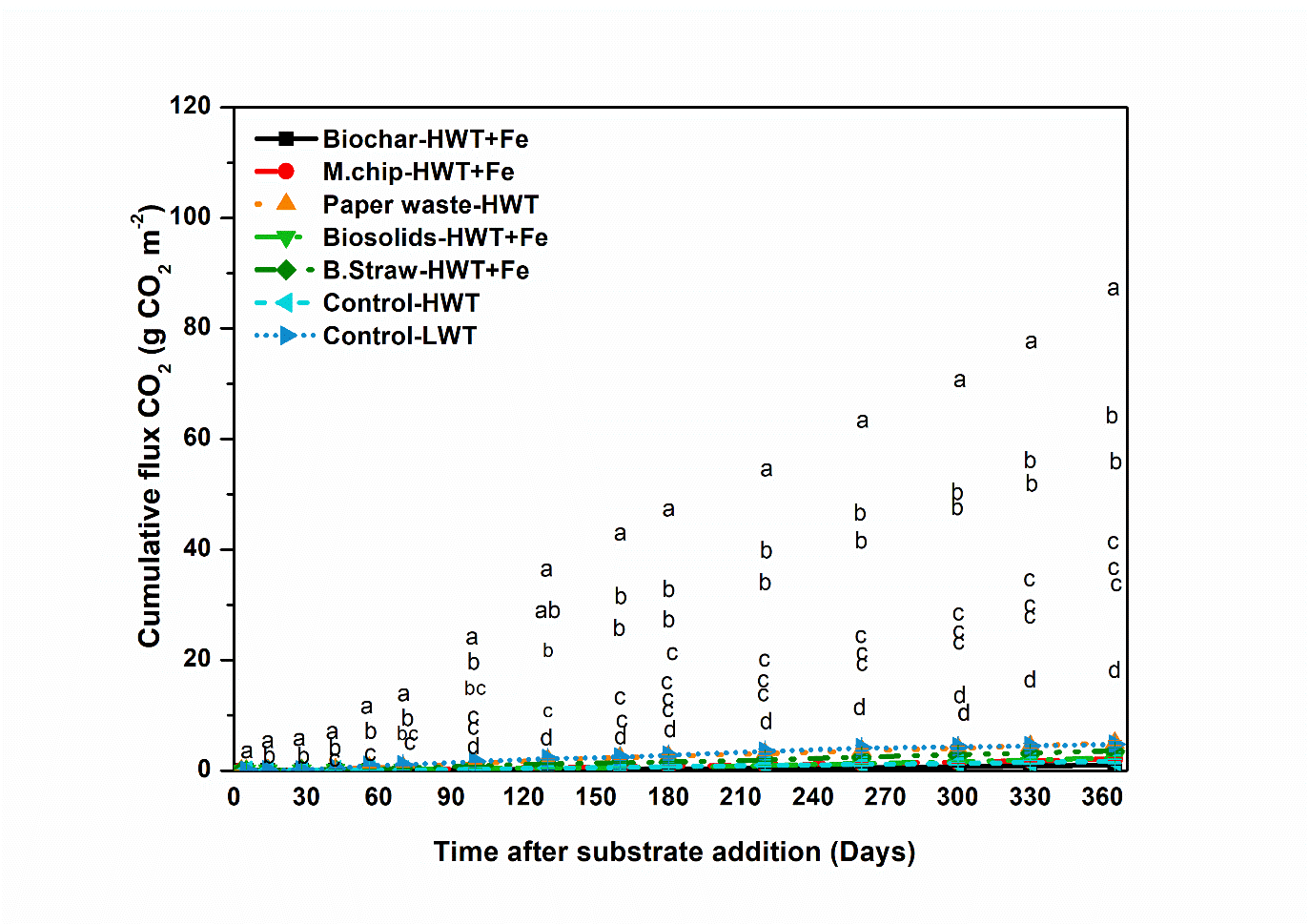

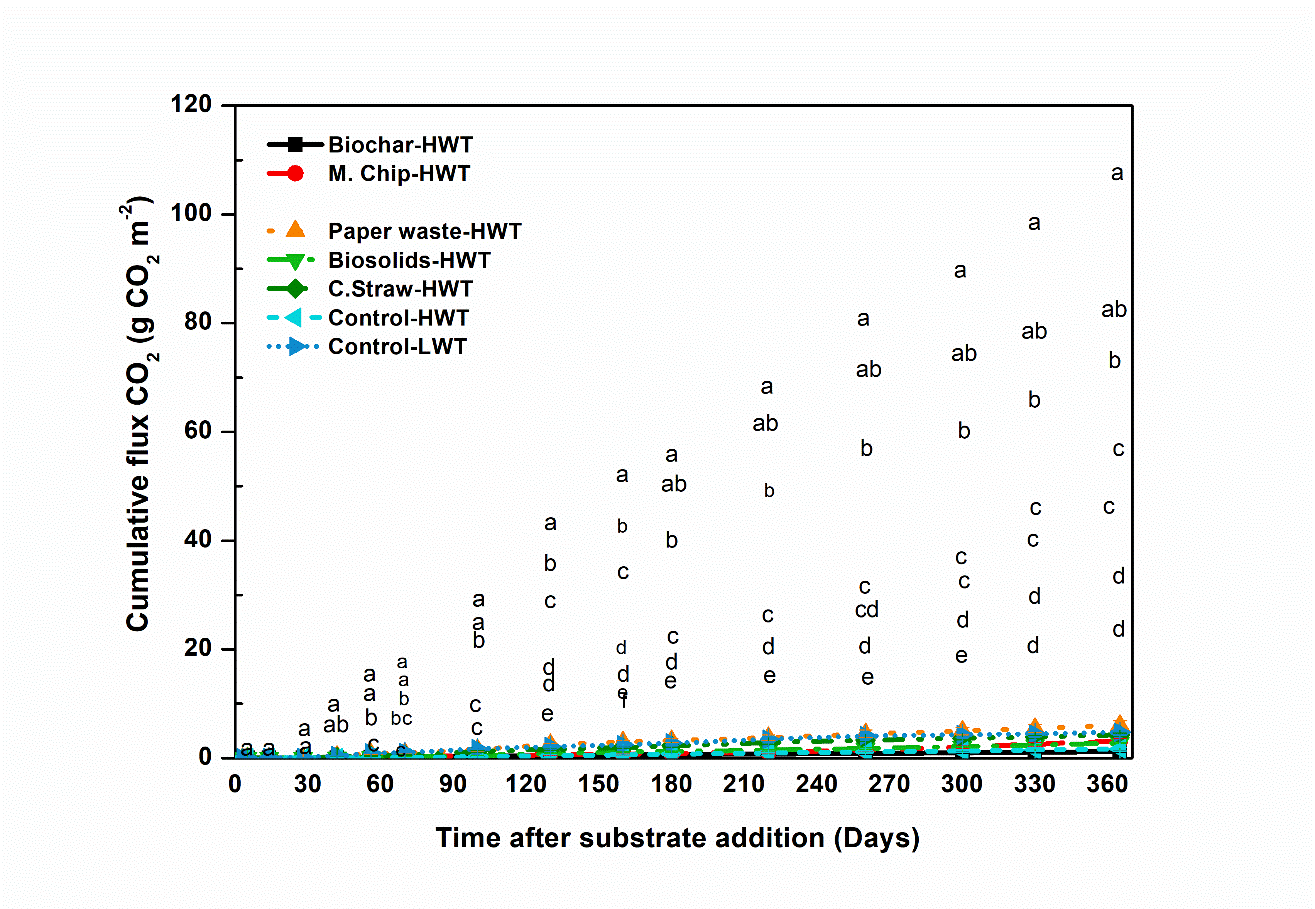

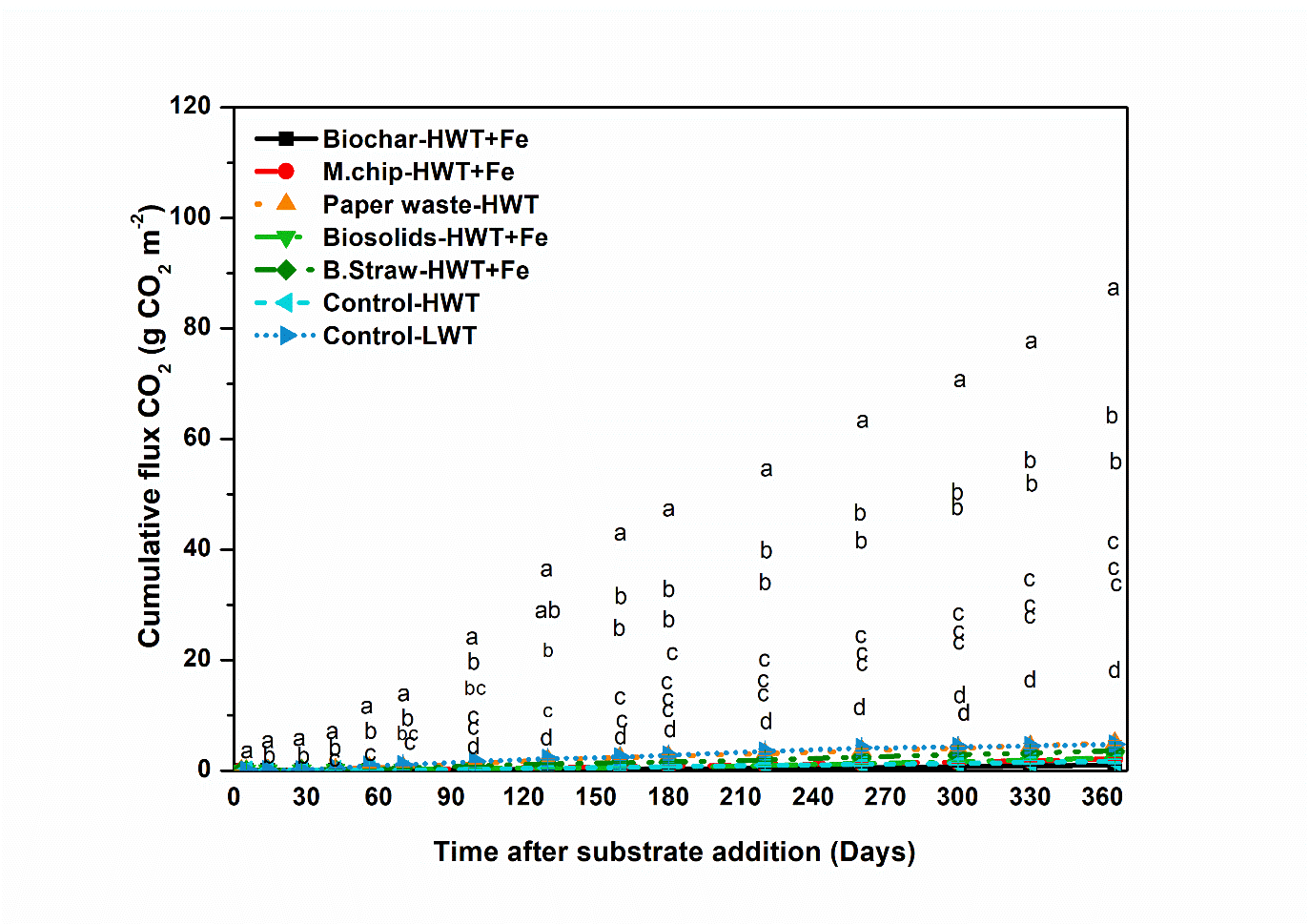


**Fig. S4** Effect of organic amendment and FeSO_4_ addition on the carbon balance in an agricultural peat soil (panels a and b). The organic amendments included *Miscanthus* biochar (Biochar), *Miscanthus* chips (M.chip), paper waste, biosolids and barley straw (B.straw). The experiment had two control consisting of a low water table (LWT) treatment and high water table (HWT) without organic amendement with and without FeSO_4_ addition. Values represent mean ± standard errors (*n* = 4).
